# Common DNA methylation dynamics in endometriod adenocarcinoma and glioblastoma suggest universal epigenomic alterations in tumorigenesis

**DOI:** 10.1038/s42003-021-02094-1

**Published:** 2021-05-21

**Authors:** Jennifer A. Karlow, Benpeng Miao, Xiaoyun Xing, Ting Wang, Bo Zhang

**Affiliations:** 1 The Edison Family Center for Genome Sciences and Systems Biology, Department of Genetics, Washington University School of Medicine, St. Louis, MO USA; 2 Center of Regenerative Medicine, Department of Developmental Biology, Washington University School of Medicine, St. Louis, MO USA; 3 McDonnell Genome Institute, Washington University School of Medicine, St. Louis, MO USA

**Keywords:** DNA methylation, Endometrial cancer, CNS cancer

## Abstract

Trends in altered DNA methylation have been defined across human cancers, revealing global loss of methylation (hypomethylation) and focal gain of methylation (hypermethylation) as frequent cancer hallmarks. Although many cancers share these trends, little is known about the specific differences in DNA methylation changes across cancer types, particularly outside of promoters. Here, we present a comprehensive comparison of DNA methylation changes between two distinct cancers, endometrioid adenocarcinoma (EAC) and glioblastoma multiforme (GBM), to elucidate common rules of methylation dysregulation and changes unique to cancers derived from specific cells. Both cancers exhibit significant changes in methylation over regulatory elements. Notably, hypermethylated enhancers within EAC samples contain several transcription factor binding site clusters with enriched disease ontology terms highlighting uterine function, while hypermethylated enhancers in GBM are found to overlap active enhancer marks in adult brain. These findings suggest that loss of original cellular identity may be a shared step in tumorigenesis.

## Abstract (web summary)

Jennifer Karlow et al. characterize the DNA methylation changes in two unrelated cancer types. They find that both cancers show methylation changes in regulatory regions corresponding to the cancer’s tissue of origin, suggesting that loss of cellular identity may be an important step in tumorigeneses shared across cancers.

## Introduction

Since the advent of the first reference human genome in 2001^1^, the identification of genetic mutations in cancer has largely established cancer as a genetic disease. Many groups have compared the mutational landscape across different cancer types to identify functional genomic mutations and pathways mechanistically linked to cancer-type-specific and pan-cancer tumorigenesis^2–8^. The more recent identification of epigenetic alterations in cancer has revealed increased complexity of cancer gene regulation, extending the view of cancer abnormalities beyond simply genetic alterations^5,9,10^.

One epigenetic modification in particular, DNA methylation, has long been associated with gene expression, where promoter methylation absence and high gene body methylation positively correlate with gene expression^11–13^. Studies regarding DNA methylation changes and additional epigenetic modifications have highlighted important roles these alterations play in cancer, leading the field to now recognize cancer as both a genetic and epigenetic disease^9,10,14–19^. DNA methylation alterations, in particular genome-wide loss and local gains within promoters, are considered hallmarks of many cancers^9,15–19^ and could possibly be causal^20–22^. As DNA methylation can impact gene expression, methylation alterations in cancer likely impact the tumor phenotype by modulating the regulatory landscape, thereby helping shape the cancer’s cell fate^10,23^. Although epigenetic abnormalities have been observed in many cancers, their specific functions and possible roles in tumorigenesis remain unclear. Moreover, commonalities in the locations of abnormal DNA methylation residing outside promoters and their functional roles across different cancer types remain understudied, as the majority of pan-cancer DNA methylation analyses to date have included only array-based methylation data^2,5,10,24^.

We have previously generated global DNA methylation profiles for endometrial cancers^25^ as well as for glioblastoma multiforme (GBM)^26^, revealing altered DNA methylation over regulatory enhancers and promoters, as well as hypomethylated gene body promoters, respectively. In addition, studies of colon cancer^27–29^ and of multiple cancer cell lines^30,31^ suggest that enhancer methylation is drastically altered in cancers and is closely related to altered transcriptional profiles. Together, these results suggest that the regulatory landscape in cancer may be altered directly, through methylation changes within regulatory elements, or indirectly, through reassignment of regulatory element target genes.

The compilation of reference human epigenomes for a variety of cell types and tissues allows for a comprehensive, cell-type-specific annotation of epigenetic abnormalities^32^. We hypothesize that by comparing the global epigenetic abnormalities of two highly distinct cancer types in the context of non-malignant cell-type epigenomes, we can unveil both shared and unique epigenetic mechanisms contributing to cancer. To better understand how epigenetic abnormalities differ between cancers in both location and function, we directly compared deeply profiled DNA methylomes of two distinct cancer types, GBM and endometrioid adenocarcinoma (EAC), whose DNA methylation abnormalities have been previously identified^25,26^. GBM, also known as grade IV astrocytoma, originates from astrocytes and quickly develops into highly heterogeneous malignancies^33^. Roughly 90% of GBM cases are classified as primary GBM and are associated with a poor clinical outcome^33^, contributing to an overall 5-year survival rate of about 5.5%^34^. Uterine corpus cancer, of which 90% of cases originate in the endometrium^35^, is associated with a much better prognosis^36^. Endometrial cancer is broadly classified into two categories. Type 1 tumors, which include EAC, constitute the majority of cases, are typically low grade, only moderately differentiated, and are hormone-sensitive^37–39^. If detected in an early, localized stage, the 5-year survival rate for uterine corpus cancers can be as high as 85–96%^36^. GBM and EAC appear to have little in common, suggesting they are good candidates for identifying shared and unique epigenetic abnormalities with predicted functional impacts on cancer phenotype.

In this study, we directly compare the DNA methylation abnormalities of these two cancer types and annotate their likely impacts on gene regulation using normal reference epigenomes. Our results indicate that both cancer types exhibit thousands of local, recurrent DNA methylation abnormalities, in the form of both increased (hyper) and decreased (hypo) methylation, which are significantly enriched in genomic regions annotated as regulatory elements, such as promoters and enhancers. Only ~50% of these DNA methylation abnormalities fall within regions targeted by the Infinium 450k array, the most common platform for DNA methylation profiling chosen by projects including The Cancer Genome Atlas (TCGA), highlighting the importance of whole-genome, unbiased approaches for profiling cancer DNA methylomes. Despite being very distinct diseases, EAC and GBM share a significant number of differentially methylated regions (DMRs). Notably, both cancers demonstrate an enrichment of hyperDMRs within the apoptosis pathway and hypoDMRs within the long terminal repeat (LTR) retrotransposon subfamily MER52A, alluding to potential pan-cancer signatures. We further report that clusters of enhancer hyperDMRs in EAC defined by the presence of binding sites for enriched TFs most notably enrich for uterine disease ontology. In addition, enhancer hyperDMRs in GBM largely overlap active enhancer histone modifications in adult brain tissue. These results suggest that regulatory regions of genes involved in functions related to the cancer’s original cell type often become methylated during tumor progression, perhaps contributing to the loss of a phenotypic cellular identity experienced by cancer cells.

Taken together, our results support findings that cancer is a complex disease with a large epigenetic component. Although the locations of DNA methylation abnormalities are often specific to a particular cancer type, many appear to functionally contribute in similar ways by potentially silencing cell-type-of-origin enhancers. Our results begin to shed light on the mechanistic principles that drive both common and cancer-type-specific DNA methylation abnormalities and their functional consequences.

## Results

### Comparative analysis of EAC and GBM DNA methylation abnormalities

We previously profiled the DNA methylomes of EAC^25^ and GBM^26^. A combined technique of methylated DNA immunoprecipitation sequencing (MeDIP-seq) and methylation-sensitive restriction enzyme sequencing (MRE-seq), which detect methylated CpGs and unmethylated CpGs, respectively, was used for methylome profiling^40^. Complete DNA methylomes were generated for a total of five GBM samples^26^, two normal frontal cortex brain samples^26^, three EAC samples^25^, and one normal endometrial sample pooled from ten healthy individuals^25^. DMRs between EAC samples and pooled normal endometrium (EAC DMRs), and between GBM samples and normal brain (GBM DMRs) were identified by integrating the MeDIP-seq and MRE-seq data using M&M^41^, allowing for the comparison of two distinct cancer-type DMR sets in the context of their normal, tissue-specific DNA methylomes. Examination of DMRs called across tumors within a specific cancer type revealed considerable intertumoral heterogeneity. However, roughly 60% of DMRs identified in one sample were also discovered in an additional sample (Supplementary Fig. 1), suggesting that the requirement for a DMR to be identified in at least two tumors to be considered, as in previous analyses^25,26^, allowed for the identification of the majority of common epigenetic changes for the given cancer type. DMRs were further classified according to their direction of methylation change, a hypermethylated DMR (hyperDMR) being more highly methylated in the cancer than in the normal, and a hypomethylated DMR (hypoDMR) being less methylated. DMRs were also classified as either cancer-type unique (DMR present in only one of the two cancers) or shared between EAC and GBM, resulting in 6 categories: EAC-unique hyperDMRs, EAC-unique hypoDMRs, GBM-unique hyperDMRs, GBM-unique hypoDMRs, shared hyperDMRs, and shared hypoDMRs.

**Fig. 1:**
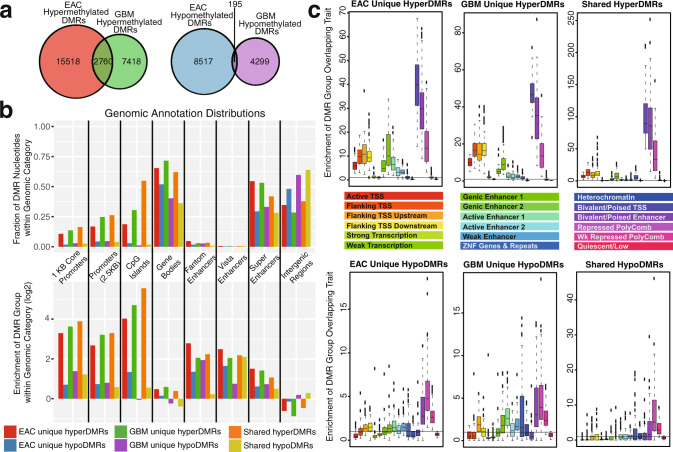
Comparative analysis of EAC and GBM DNA methylation abnormalities. **a** Overlap of 500 bp differentially methylated regions (DMRs) in EAC and GBM. Left: hypermethylated DMRs; right: hypomethylated DMRs. Both overlaps are significant (*p* < 2.2E − 308 and *p* < 6.0E − 199, respectively, hypergeometric tests). **b** Genomic annotation distribution of EAC and GBM DMRs. Top: percentage of DMR group nucleotides within each genomic category. Bottom: enrichment of DMR group nucleotides within each genomic category. **c** EAC and GBM DMR enrichment within epigenetic annotations (chromHMM 18-states) across a variety of cell and tissue types (*n* = 80, Human Roadmap Epigenome^32^). For each DMR group, the percentage of base pairs (bps) that overlapped each epigenetic state in each of the cell/tissue types was calculated, and enrichment scores were calculated by dividing those percent overlaps by the percentage of background bps that overlapped each epigenetic state in each cell/tissue type. Background regions considered when calculating enrichment in both **b** and **c** were 500 bp genomic regions that excluded chromosome ends, excluded bins overlapping blacklisted CpGs, excluded bins not containing at least one MeDIP and/or MRE read for at least one sample, excluded bins not containing at least one CpG, and excluded those on chrY (and chrX for GBM).

We identified 26,990 DMRs in EAC (10,414 (38.6%) of which were shared across all 3 samples) and 14,672 in GBM (426 (2.9%) of which were shared across all 5 GBM samples). The ratio of hyperDMRs to hypoDMRs was remarkably similar between GBM (ratio = 2.265 : 1) and EAC (ratio = 2.098 : 1). Although the number of shared hyperDMRs (*n* = 2760) drastically outnumbered the shared hypoDMRs (*n* = 195), both were highly significant (*p* < 2.2E − 308 and *p* < 6.0E − 199, respectively, hypergeometric tests, using as background 500 bp genomic regions with at least one CpG and MeDIP and/or MRE signal in at least one sample) (Methods and Fig. 1a). Examination of the methylation levels of the identified shared EAC and GBM DMR regions within TCGA samples spanning 24 cancer types revealed similar trends (Methods). Specifically, 22/24 cancer types profiled in TCGA with both tumor and normal methylation data showed a significant increase in methylation from normal to tumor over shared EAC and GBM hyperDMRs (Supplementary Fig. 2). Similarly, 21/24 cancer types profiled in TCGA exhibited a significant decrease in methylation over shared EAC and GBM hypoDMRs (Supplementary Fig. 3), supporting that the methylation changes observed over these shared regions likely extend beyond EAC and GBM, possibly corresponding to a pan-cancer methylation signature.

To better understand the possible functional contribution of altered DNA methylation within EAC and GBM, we identified the fraction as well as enrichment of DMRs that fell into genomic regions annotated as promoters (1 kb and 2.5 kb), CpG islands, gene bodies, enhancers defined either by Fantom 5^42,43^, or by the VISTA enhancer project^44^, super enhancers^45^, and intergenic regions. We found that the three hyperDMR groups (EAC-unique hyperDMRs, GBM-unique hyperDMRs, and shared hyperDMRs) exhibited a much higher percentage overlap to promoter regions, CpG islands, gene bodies, and super enhancers than did the three hypoDMR groups (EAC-unique hypoDMRs, GBM-unique hypoDMRs, and shared hypoDMRs), also reflected by increased relative enrichment (Fig. 1b). Conversely, the hypoDMR groups exhibited a higher percentage overlap to intergenic regions, where the hyperDMR groups and EAC-unique hypoDMRs were depleted within intergenic regions (Fig. 1b). The high enrichment of both hyperDMRs and hypoDMRs in Fantom and VISTA enhancers suggests that *cis*-regulatory enhancer elements may contain both activating and inactivating DNA methylation abnormality hotspots in cancer.

DMRs were further characterized according to their chromatin-state annotations, defined based on various histone modifications^32^. Taking advantage of the 18-state chromatin models (chromHMM) generated from complete human epigenome references for various cell and tissue types^46^, we calculated the fraction and enrichment of DMRs overlapping each chromatin state across many different cell and tissue types (*n* = 80) (Methods). HyperDMR groups were generally enriched within regulatory regions annotated as active transcription start sites (TSSs), regions flanking TSSs (both upstream and downstream), genic enhancers, and active enhancers across different tissues (Fig. 1c and Supplementary Fig. 4). In contrast, hypoDMRs exhibited a range of enrichment values in active chromatin states, varying from depletion to weak enrichment (Fig. 1c and Supplementary Fig. 4). Although hyperDMRs were most highly enriched within bivalent/poised TSSs across tissues, hypoDMRs were generally depleted or had little enrichment over these regions. Conversely, hypoDMRs were enriched within weakly repressed polycomb regions across tissues, whereas hyperDMRs were generally depleted or showed no enrichment (Fig. 1c and Supplementary Fig. 4). HyperDMRs and hypoDMRs were both enriched within repressed polycomb regions and bivalent/poised enhancers, although the magnitude of enrichment was much higher for hyperDMRs in both cases. In concordance with the most severe gain of methylation in both cancer types residing over polycomb and bivalent regions, we found that EZH2 binding in normal human astrocytes^47^ was highly enriched within GBM hyperDMRs, suggesting that these tumors undergo an epigenetic switch involving gain of DNA methylation over regions normally bound by EZH2 in non-malignant astrocytes (Supplementary Table 1). These results demonstrate that DMRs are enriched within regulatory regions in both cancers, both within and outside promoters, highlighting the complexity of DNA methylation alterations within cancer^27–31^.

### EAC and GBM DMRs exhibit similar characteristics on a pathway level

**Fig. 2:**
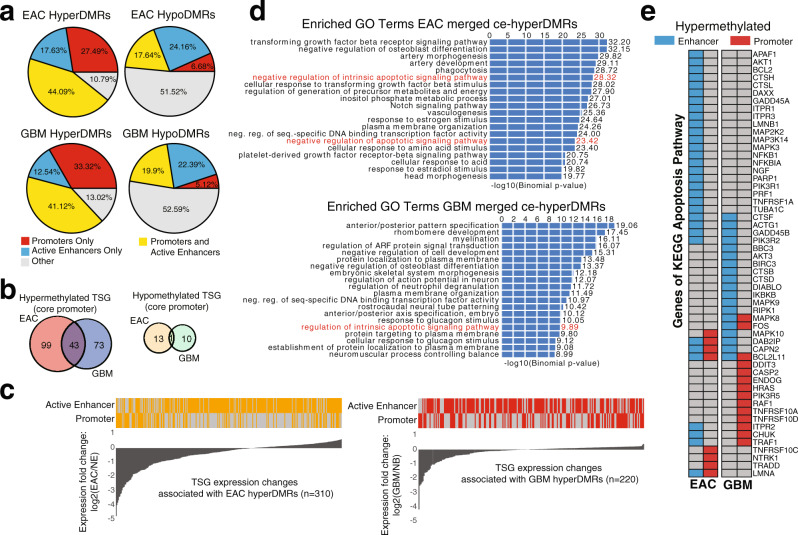
EAC and GBM DMRs show similar characteristics on a pathway level. **a** Percentage of DMRs with active regulatory annotations (promoter and/or active enhancer) in EAC and GBM. **b** Overlap of tumor suppressor genes (TSGs) with abnormally methylated core promoters (1 kb, centered around TSS) in EAC and GBM. Left: hyperDMRs (overlap is significant: *p* < 8.39E − 14, hypergeometric test); right: hypoDMRs. **c** TSGs with abnormally methylated promoters (1 kb, centered around the TSS) and/or active enhancers in EAC (left) and GBM (right), accompanied by fold change in expression of TSGs in EAC and GBM. NB: normal brain; NE: normal endometrium. Orange and red bars indicate the presence of a DMR within an active enhancer/promoter in EAC and GBM hyperDMRs, respectively. **d** Top enriched GO terms (Biological Process) of ce-hyperDMRs in EAC (top) and GBM (bottom). Values represent the −log10(Binomial *p*-value). **e** Hypermethylation status of apoptosis gene promoters (red) and enhancers (blue) in EAC (left) and GBM (right).

To better understand the shared functionality of DMRs between EAC and GBM, we compared the frequency at which they contained potential regulatory regions. We found that although only about half of all EAC and GBM hypoDMRs overlapped regions with regulatory annotations, this percentage increased to roughly 90% for EAC and GBM hyperDMRs, reflecting a large enrichment within both cancers. We found that EAC and GBM hyperDMRs largely overlapped regions annotated as both promoters and enhancers (44.09% and 41.12%, respectively), whereas an additional 27.49% and 33.32% overlapped promoters only, and an additional 17.63% and 12.54% overlapped active enhancers only (Methods and Fig. 2a).

As the silencing of tumor suppressor genes (TSGs) by DNA methylation is a common mechanism of gene inactivation in cancer^20–22,48–50^, we examined the frequency at which DMRs overlapped TSG core promoters. We found that the number of TSGs suffering hypermethylation within their core promoters in EAC and GBM, as well as those overlapping shared hyperDMRs, were statistically significant (*p* < 2.22E − 08, *p* < 5.27E − 05, and *p* < 1.94E − 03, respectively; hypergeometric tests) (Supplementary Data 1). The number of TSGs suffering hypomethylation within their core promoters was not significant for either EAC or GBM individually, or for those overlapping shared hypoDMRs (Supplementary Data 1). In addition, the number of TSGs undergoing hypermethylation in both EAC and GBM was higher than expected by chance (*p* < 8.39E − 14; hypergeometric test), suggesting that methylation of some of the same TSGs might be a shared attribute of these cancers (Fig. 2b and Supplementary Data 2).

As enhancers frequently undergo DNA methylation changes during tumorigenesis, we further examined distal DMRs near TSGs. Hypermethylation in promoters and/or active enhancers was found in 350 TSGs in EAC and 245 TSGs in GBM (Fig. 2c and Supplementary Data 3). Hypermethylation of regulatory elements around TSGs was generally associated with decreased expression of TSGs in these two cancer types.

We next examined the commonalities in distal DNA methylation changes between these two cancer types on a pathway level. In our study, the majority of EAC and GBM DMRs fell outside gene promoters; however, many of these non-promoter DMRs (EAC hyperDMRs: 72%, GBM hyperDMRs: 63%, EAC hypoDMRs: 46%, GBM hypoDMRs: 45%) exhibited an active enhancer signature in at least one of the 80 different tissues or cell types provided by Roadmap Epigenomics Consortium^32^. Therefore, we defined non-promoter DMRs that fell within regions annotated as an active enhancer (state “9_EnhA1” or “10_EnhA2”) in at least 1 of 80 epigenomes as cancer-enhancer DMRs (ceDMRs). By comparing enriched biological processes associated with merged ceDMRs (Methods), we found that both cancer-type cancer-enhancer hyperDMRs (ce-hyperDMRs) were highly enriched for terms related to apoptosis, a process commonly deregulated in cancer cells^51^, sometimes through DNA methylation alterations^52^ (Fig. 2d and Supplementary Data 4). In EAC, 36 of the 140 apoptosis-associated genes contained hyperDMRs within regulatory elements, 4 of which contained hyperDMRs over both promoters and enhancers. Similarly, in GBM, 30 of the 140 apoptosis-associated genes contained regulatory element hyperDMRs, 3 of which exhibited both promoter and enhancer hyperDMRs (Fig. 2e). One gene in particular, *BCL2L11*, contained a single hyperDMR that spanned both the gene’s promoter and an active enhancer in both cancer types (Supplementary Fig. 5). Although both EAC and GBM hyperDMRs were enriched within apoptosis pathway genes, only 11 genes gained regulatory region methylation in both cancer types (Fig. 2e), highlighting the complex strategies different cancers might take to reach the same functional consequences.

### Abnormally methylated enhancer-potential regions in cancer are associated with deregulated TFs

**Fig. 3:**
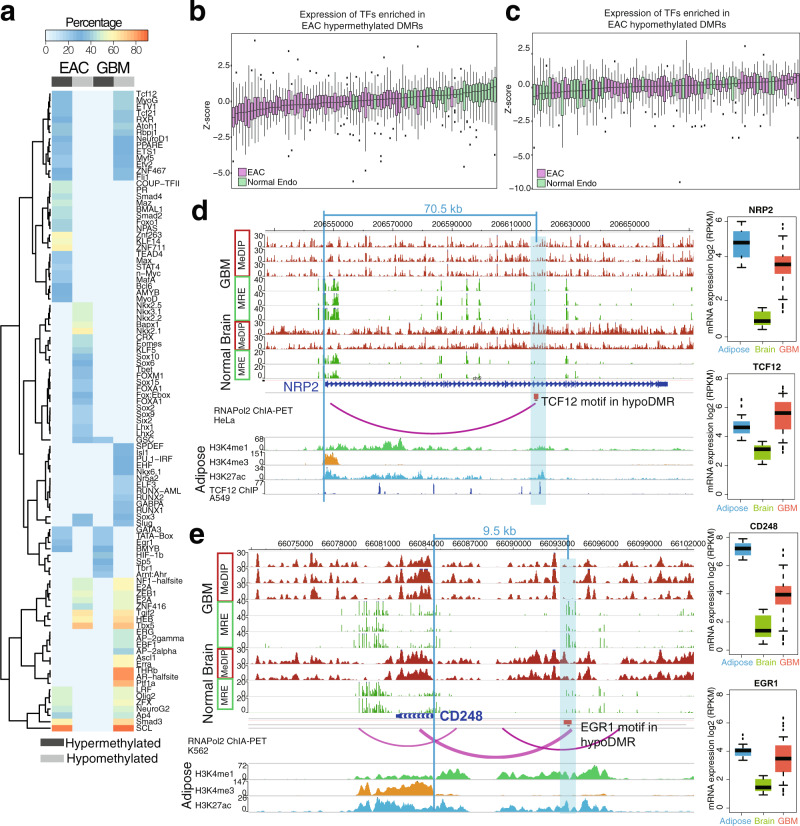
Abnormally methylated enhancer-potential regions in cancer are associated with deregulated transcription factors. **a** Percentage of EAC and GBM DMRs with enriched transcription factor-binding motifs. Only those with a *q*-value ≤ 0.01 and percentage ≥ 20% are shown. **b**, **c** Normalized expression (RPKM, *z*-scores) of TFs enriched in EAC ce-hyperDMRs (**b**, number of EAC samples = 57, number of normal endometrial samples = 29, number of TFs = 30) and ce-hypoDMRs (**c**, number of EAC samples = 57, number of normal endometrial samples = 29, number of TFs = 21) in normal endometrium (green) and EAC (purple) samples. **d** Epigenome Browser^74,75^ view demonstrating a hypoDMR (blue highlighted region) across GBM samples within the *NRP2* gene. The DMR contains a TCF12-binding motif, as evidenced by a ChIP-seq peak for the TF in A549 cells, which demonstrates interaction with the *NRP2* promoter in HeLa cells. *TCF12* is relatively unexpressed in brain (frontal cortex, *n* = 28), but more highly expressed in adipose tissue (subcutaneous, visceral, *n* = 1204) where it also bears the marks of an active enhancer (H3K4me1 and H3K27ac, lacking H3K4me3). Expression of *TCF12* increases in GBM (*n* = 160), accompanying an increase in the presumed target gene *NRP2*’s expression, as also seen in adipose tissue. MeDIP and MRE tracks depict raw read data. **e** Epigenome Browser^74,75^ view demonstrating a hypoDMR (blue highlighted region) across GBM samples upstream of the *CD248* gene. The DMR contains an EGR1-binding motif that demonstrates interaction with the *CD248* promoter in K562 cells. *EGR1* is relatively unexpressed in brain (frontal cortex, *n* = 28), but more highly expressed in adipose tissue (subcutaneous, visceral, *n* = 1204) where it also bears the marks of an active enhancer (H3K4me1 and H3K27ac, lacking H3K4me3). Expression of *EGR1* increases in GBM (*n* = 160), accompanying an increase in the presumed target gene *CD248’*s expression, as also seen in adipose tissue. MeDIP and MRE tracks depict raw read data.

DNA cytosine methylation status has been shown to be associated with transcription factor (TF)-binding events^53,54^, although the mechanism linking the two remains unclear. Motif discovery within ceDMRs identified highly enriched sets of TF-binding sites (TFBSs; Fig. 3a), most of which were exclusive to one cancer type. Expression of TFs with motifs enriched in EAC ce-hyperDMRs were downregulated in EAC compared to normal endometrium (Fig. 3b), whereas TFs whose bindings motifs were enriched in EAC cancer-enhancer hypoDMRs (EAC ce-hypoDMRs) were generally upregulated in EAC (Fig. 3c), suggesting that changes in TF expression may help dictate changes in DNA methylation at target motifs in EAC.

In support of this directionality, we observed cases of hypomethylation in GBM over non-brain enhancers, accompanied by gain in expression of both the TFs with predicted binding motifs and their target genes, illustrated in two examples (Fig. 3d, e). Neuropipin-2 (NRP2), a non-tyrosine kinase receptor frequently overexpressed in various malignancies, including GBM, regulates endosome maturation and EGFR trafficking, supporting the growth and replication of cancer cells^55^. We identified a GBM hypoDMR in the *NRP2* gene body, located 70.5 kb downstream of the *NRP2* TSS (Fig. 3d). RNA polymerase II ChIA-PET data in HeLa cells generated by the ENCODE consortium indicated a direct physical interaction between the *NRP2* TSS and the hypoDMR in this particular cell line, arguing that these two genomic regions have the potential to interact. In addition, this region also contained strong H3K27ac and H3K4me1 signal in adipose tissue, indicative of an active enhancer in this cell type. Motif analysis revealed the presence of a TCF12-binding site within this hypoDMR and chromatin immunoprecipitation sequencing (ChIP-seq) data in a cancer cell line (A549) supported strong binding of TCF12 in this enhancer region. *TCF12* and *NRP2* were found to be relatively highly expressed in adipose tissue, lowly expressed in the brain frontal cortex, and highly expressed again in the GBM. Therefore, this site reflects a possible co-opted adipose enhancer that lost DNA methylation in GBM cells, possibly as a result of abnormal upregulation of *TCF12* in GBM, resulting in an upregulation of *NRP2*. Similarly, CD248 (Endosailin) marks tumor-associated pericytes in high-grade glioma^56^, where blocking *CD248* can inhibit the growth and differentiation of perivascular cells^57^. We identified a GBM hypoDMR located ~9 kb upstream of *CD248*, which contained an enhancer with strong H3K27ac and H3K4me1 signal in adipocyte tissue (Fig. 3e). K562 RNA polymerase II ChIA-PET data suggested this enhancer has the capability to physically interact with the *CD248* TSS. An EGR1-binding motif was identified within this hypoDMR and both *EGR1* and *CD248* were more highly expressed in adipose and GBM than in normal brain. This suggests another example where GBM is adopting the potential regulation of *CD248* by *EGR1* seen in adipose tissue.

### Gain of methylation over original cell-type enhancers may contribute to loss of cellular identity during cancer progression

**Fig. 4:**
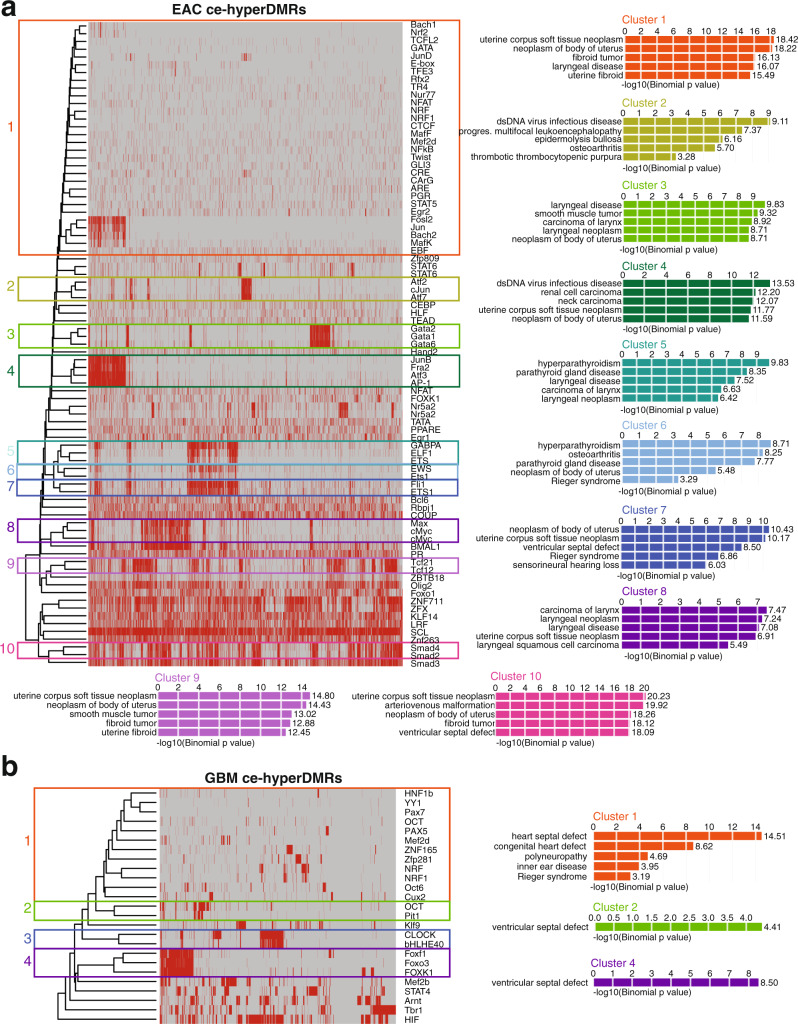
Gain of methylation over original cell-type enhancers may contribute to loss of cellular identity during cancer progression. **a** Heatmap indicating the presence of enriched TF-binding motifs in EAC ce-hyperDMRs and top GO disease ontology enrichment results for DMR clusters (red: present; gray: absent). **b** Heatmap indicating the presence of enriched TF-binding motifs in GBM ce-hyperDMRs and top GO disease ontology enrichment result for DMR clusters (red: present; gray: absent).

Although DNA methylation alterations in GBM and EAC exhibited many commonalties, the two cancers also displayed distinct signatures, reflecting tissue type specificity^5^. To better understand these unique differences, we first calculated the enriched vertebrate TF-binding motifs within the ceDMRs using Homer^58^ and opposite ceDMR groups as background (Methods). Enriched motifs were then filtered to only include those TFs with significant expression changes in TCGA, and ceDMRs were then clustered based on presence or absence of these enriched motifs, using a distance method of “Euclidean” and a clustering method of “complete” (Methods). Clusters of ceDMRs were identified by cutting the dendrogram at various heights, which resulted in robust groupings and enriched Gene Ontology (GO) terms. Clustering ceDMRs by the presence of enriched TF-binding motifs revealed several clusters of DMRs with similar subsets of TF-binding motifs, likely reflecting the high similarity among binding motifs of related TFs, e.g., GATA family (Cluster 3) and SMAD family (Cluster 10) in EAC ce-hyperDMRs (Fig. 4a) and the FOX family (Cluster 4) in GBM ce-hyperDMRs (Fig. 4b). However, despite a propensity for clusters of DMRs to harbor motifs for a handful of TFs, many different, sometimes largely non-overlapping clusters of EAC ce-hyperDMRs (clusters 1, 3, 4, 6, 7, 8, 9, and 10) enriched for similar disease ontology terms, most commonly centered around uterine neoplasia (Fig. 4a), suggesting that targeted silencing of intrinsic cell-identity pathways may contribute to cancer progression. GBM ce-hyperDMR clusters of enriched TF-binding motifs, on the other hand, enriched for terms most notably related to heart functions (Fig. 4b).

**Fig. 5:**
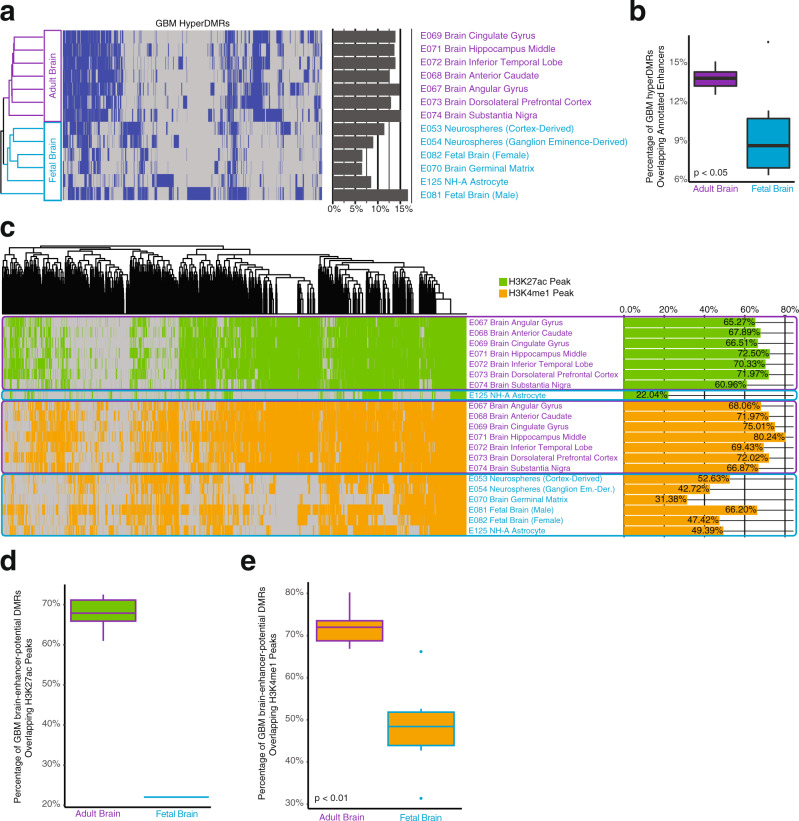
GBM hyperDMRs possess enhancer chromatin marks in adult brain tissues. **a** Enhancer annotation (chromHMM 15-state model annotation “7_Enh”^32^) presence or absence in GBM hyperDMRs in fetal (*n* = 6, light blue) and adult brain (*n* = 7, purple) tissue/cells^32^ (heatmap colors: blue: enhancer annotation present in DMR; gray: enhancer annotation absent in DMR). Bar plot indicates the fraction of all GBM hyperDMRs overlapping enhancer annotations in each cell/tissue type. **b** Boxplot depicting the distribution of fractions of GBM hyperDMRs overlapping enhancer annotations, comparing adult (*n* = 7) and fetal (*n* = 6) brain tissues (*t*-test, *p* < 0.05). **c** H3K27ac (green) and H3K4me1 (orange) peak occupancy within GBM enhancer-potential hyperDMRs (GBM hyperDMRs overlapping an enhancer annotation in at least one adult or fetal brain tissue) across fetal (*n* = 1 and 6, for H3K27ac and H3K4me1, respectively, light blue) and adult (*n* = 7 for both H3K27ac and H3K4me1, purple) brain tissues. Bar plot indicates the fraction of all GBM enhancer-potential hyperDMRs that contained H3K27ac peaks or H3K4me1 peaks in each cell/tissue type. **d** Boxplot depicting fractions of GBM enhancer-potential hyperDMRs overlapping H3K27ac peaks in adult brain tissues (*n* = 7, left) and horizontal line depicting the fraction of GBM enhancer-potential hyperDMRs overlapping H3K27ac peaks in fetal brain (*n* = 1, right). **e** Comparison of the proportions of GBM enhancer-potential hyperDMRs overlapping H3K4me1 peaks in adult brain tissues (*n* = 7, left) to the proportions of GBM enhancer-potential hyperDMRs overlapping H3K4me1 peaks in fetal brain tissues (*n* = 6, right) (*t*-test, *p* < 0.01).

To better understand whether brain functionality was being targeted by hypermethylation in GBM, we classified whether distal GBM hyperDMRs (those located outside promoters) overlapped the enhancer-defined chromatin state (“7_Enh” based on the chromHMM 15-state model) for 13 adult and fetal brain-related tissues using the brain epigenome references generated by the Roadmap Epigenomics Consortium^32^. We found that a significantly greater proportion of GBM hyperDMRs overlapped annotated enhancers in adult brain tissues as opposed to in fetal astrocyte and progenitor cells, with the exception of the male fetal brain sample (Fig. 5a, b). To characterize the potential activity of brain enhancers that gained methylation in GBM, we determined whether they overlapped ChIP-seq peaks for H3K27ac and H3K4me1 in fetal and adult brain tissues (Methods). We found that the majority of GBM hyperDMR brain enhancers overlapped H3K27ac peaks in adult brain (60.96–72.50%), whereas very few GBM hyperDMR brain enhancers overlapped H3K27ac peaks in fetal brain (22.04%) (Fig. 5c, d). The percentage of H3K27ac peaks overlapping GBM hyperDMR brain enhancers was much greater in the adult brain samples (0.725–0.877%) compared to the fetal brain sample (0.314%), suggesting that the increased number of GBM hyperDMR brain enhancers overlapping H3K27ac peaks in adult brain is not due to increased global H3K27ac signal, but is in fact specific. Likewise, the majority of GBM hyperDMR brain enhancers overlapped H3K4me1 peaks in adult brain (66.87–80.24%), whereas a significantly smaller proportion overlapped H3K4me1 peaks in fetal brain (31.38–66.20%) (Fig. 5c, e). In addition, although the percentage of H3K4me1 peak base pairs within GBM hyperDMR brain enhancers was similar between adult and fetal brain samples (0.68–1.01% vs. 0.463–0.803%, respectively), a higher percentage overlap in the adult brain samples indicates the increase in H3K4me1 signal in adult brain is not simply due to increased background H3K4me1 signal. These results suggest that although a portion of GBM hyperDMR brain enhancers bear the mark of active enhancers (H3K4me1) in the developing brain, a significantly greater percentage contain these marks in adult brain tissues. In addition, most of these enhancers are not located in open chromatin (H3K27ac peaks) in developing tissue, suggesting they might be more active in adult tissue and, therefore, important for the maintenance of normal functionality of mature glia cells as opposed to brain and glial cell development.

**Fig. 6:**
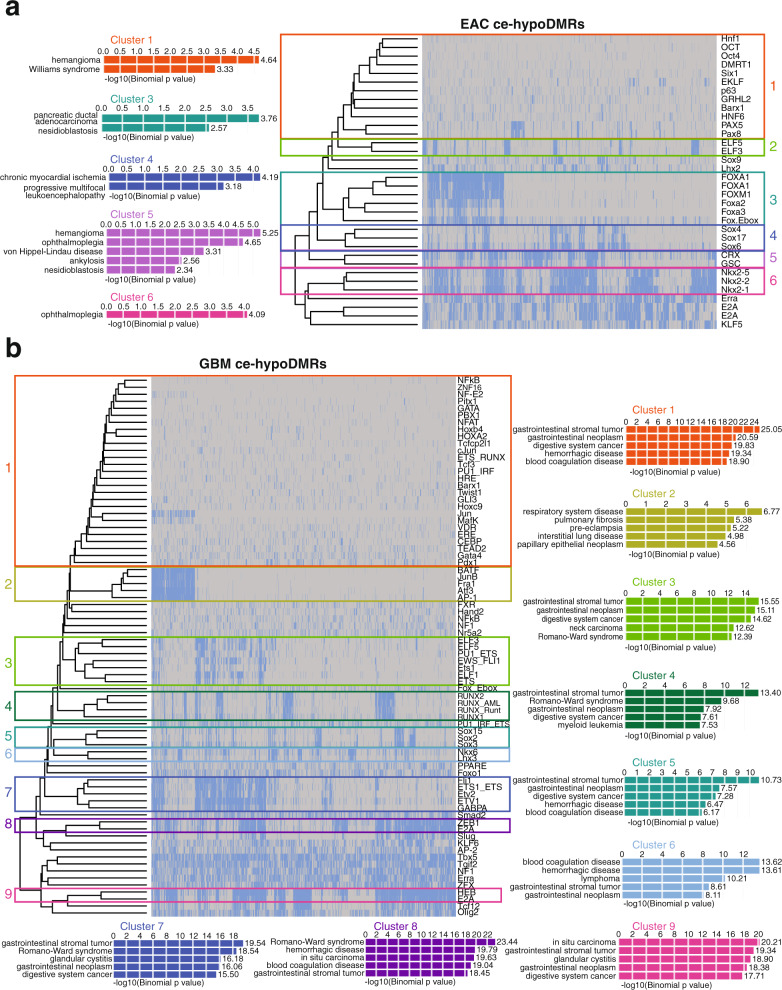
Shared loss of methylation in EAC and GBM over regions encompassing cancer-related enhancers and motifs for upregulated TFs. **a** Heatmap indicating the presence of enriched TF-binding motifs in EAC ce-hypoDMRs and top GO disease ontology enrichment results for clusters of DMRs (blue: present; gray: absent). **b** Heatmap indicating the presence of enriched TF-binding motifs in GBM ce-hypoDMRs and top GO disease ontology enrichment results for clusters of DMRs (blue: present; gray: absent).

When considering TFs with motifs enriched within EAC ce-hypoDMRs, 30 exhibited a corresponding increase in expression in TCGA EAC samples (a subset of the TCGA uterine corpus endometrial carcinoma (UCEC) cohort). Clustering these 30 TFs based on enriched motif locations within the ce-hypoDMRs revealed 6 clusters of more than 1 TF. In contrast to targeting enhancers related to the cell-type-of-origin function, EAC ce-hypoDMR clusters that contained enriched TF-binding motifs often enriched for various cancer types, most notably hemangiomas (Fig. 6a). Similarly, 67 TFs with enriched motifs within GBM ce-hypoDMRs demonstrated an increased expression in TCGA GBM samples, comprising 9 TF clusters. The majority of enrichment terms across all clusters of associated ce-hypoDMRs were related to various tumors, most notably gastrointestinal (Fig. 6b). Taken together, we observe that enhancer-potential regions with loss of methylation, containing enriched motifs for TFs that exhibit increased expression, are primarily tied to a variety of cancers, suggesting that the loss of methylation over and potential activation of aberrant enhancers may be a common trend among distinct cancer types.

### Distinct spectrum of epigenetic abnormalities within TEs in cancers

**Fig. 7:**
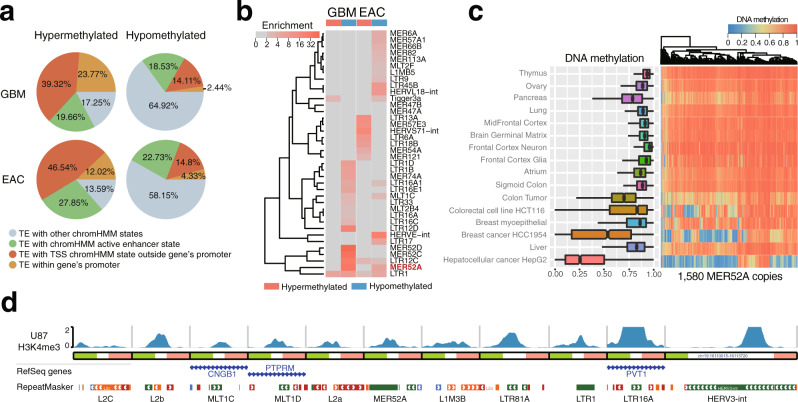
Distinct spectrum of epigenetic abnormalities within transposable elements in cancers. **a** Percentage of abnormally methylated transposable elements (TEs) within predicted regulatory regions (promoter and enhancer) in EAC and GBM. ChromHMM 18-state models^32^ were used to define active enhancer states and TSS states outside genic promoters. **b** Enrichment of abnormally methylated TEs in EAC and GBM at the subfamily level. **c** DNA methylation level of MER52A copies across different tissues and cancer types. Left: boxplot showing DNA methylation across all MER52A copies (*n* = 1580). Right: heatmap showing DNA methylation across all MER52A copies. **d** Epigenome Browser^74,75^ view of the promoter-associated histone modification H3K4me3 in the U87 cell line (GBM cells, normalized reads per million) across 11 TEs.

Transposable elements (TEs) are hotspots of epigenetic abnormalities during carcinogenesis and were generally believed to be globally hypomethylated in cancer cells^59^. We observed that 39–62% of DMRs contained TEs (GBM: 44% of hyperDMRs and 49% of hypoDMRs; EAC: 39% of hyperDMRs and 62% of hypoDMRs). Although 23.77% and 12.02% of GBM and EAC hyperDMR-overlapped TEs, respectively, fell within RefSeq-defined promoters, an additional 39.32% and 46.54%, which were located outside canonical promoters, were annotated as TSSs in at least 1 of the 80 Roadmap cell/tissue types, based on chromHMM 18-state chromatin predictions (Fig. 7a). In addition, 18.53–27.85% of DMR-overlapped TEs outside promoters fell within predicted active enhancer regions based on Roadmap Epigenomics data chromHMM 18-state chromatin predictions (Fig. 7a). We estimated the enrichment of epigenetically altered TE subfamilies and discovered distinct patterns within GBM and EAC (Fig. 7b). A small number of TE subfamilies, including LTR16A1, MLT1C, and MER52A, were highly enriched in both GBM and EAC hypoDMRs, where the primate-specific LTR retrotransposon MER52A exhibited the highest enrichment (Fig. 7b). We further examined the DNA methylation level of MER52A copies in normal tissues and cancer, and found that the majority of MER52A subfamily copies were highly methylated in various normal tissues, but became demethylated in cancer (Fig. 7c). A small number of MER52A copies were lowly methylated in normal breast myoepithelial cells, liver, and pancreas tissues, suggesting that some MER52A copies maintain an active epigenetic state and may provide regulatory functions in normal cells. Finally, we found that several TEs overlapping hypoDMRs in GBM encompassed H3K4me3 signal, a mark of active promoters, in the GBM cell line U87, consistent with previous findings of hypomethylated TEs providing cryptic promoters during tumorigenesis^60^ and embryonic development^61^ (Fig. 7d).

## Discussion

In the present study, we sought to expand the current view of methylation alteration comparisons across distinct cancer types. By utilizing DNA methylation data derived from MeDIP-seq and MRE-seq, we were able to comprehensively explore the propensity for methylation alterations in cancer to be specific, based on their cell type of origin, and the ways in which alterations were shared physically or functionally between two distinct cancer types: GBM and EAC. We identified thousands of DMRs in both cancers, highly enriched within regulatory regions including promoters and enhancers.

The Infinium 450k array platform and more recent 850k array platform have been the standard of practice for measuring methylation and the method of choice for many TCGA studies^2,5,62–64^. When validating our DMRs using cancer DNA methylation data generated by TCGA with the Infinium 450K platform, we found that roughly half of our DMRs could not be detected, due to the platform’s relatively low coverage (52.55% and 46.55%, EAC and GBM DMRs, respectively) (Supplementary Table 2). Even when comparing to the more recent 850K platform, we still found that 41.85% (EAC) and 38.13% (GBM) of our DMRs were missed (Supplementary Table 2). HypoDMRs often did not contain a probe (75.87% (450k) and 62.44% (850k) for EAC, and 81.55% (450k) and 67.00% (850k) for GBM), whereas more than half of all hyperDMRs contained probes (58.57% (450k) and 67.97% (850k) for EAC, and 68.90% (450k) and 74.62% (850k) for GBM. Forty-one to 49% of the CpGs within DMRs profiled via our method but missed by the Infinium 450K array were located within regions annotated as having active enhancer potential based on Roadmap’s chromHMM 18-state annotations. As we and others have demonstrated that methylation at enhancer regions can play an important role in cancer^25,26,65^, the inclusion of this set of CpGs not covered by the Infinium array, but covered by our method, is instrumental in understanding the impact DNA methylation abnormalities have on cancer.

Although the combined use of MeDIP-seq and MRE-seq to interrogate genome-wide methylation levels has many advantages over array-based techniques, it should be noted that this approach is not without limitations. For example, MeDIP-seq enriches for genomic regions with a methylated CpG; however, the exact CpG that is methylated within a given read captured is unknown. In addition, MRE-seq is limited to interrogate restriction sites for enzymes used in the protocol, which only cover a small fraction of all genomic CpG sites^66^. Although these methods do not produce a quantitative measurement of the methylation status at each CpG, as is generated using whole-genome bisulfite sequencing (WGBS), together these methods provide complementary data that can be used to computationally predict the methylation status of individual CpGs, which recapitulate WGBS results well and at a fraction of the cost^41^.

Upon discovering that there were many more shared hyperDMRs between the two cancer types than expected by chance, we sought to elucidate possible functions within these regions. As expected, we found that TSG core promoters were often enriched within hyperDMRs. Examination of expression changes over TSGs with enhancer and/or promoter hypermethylation revealed general expression loss within tumors compared to normals, suggesting that different cancers jointly alter the methylation status of TSG regulatory regions, possibly contributing to their loss of expression in cancer.

GBM and EAC hyperDMRs also enriched for active enhancer regions, which displayed the unique enrichment of several biological processes and pathways, as well as shared enrichment regarding processes such as apoptosis. Examination of 140 apoptosis-related genes revealed several with increased promoter methylation in both cancer types, whereas many more accrued methylation changes within the surrounding enhancers. These results suggest that silencing genes involved in the apoptotic signaling pathway via methylation at enhancers and promoters may be a common mechanism shared across cancer types.

The extent to which DNA methylation alterations shape transcriptional activity remains unclear in cancer. To better understand the relationship between altered DNA methylation and phenotypic impact via gene expression changes, we identified enriched TF-binding motifs in both EAC and GBM ceDMRs. We found that TFs associated with EAC ce-hyperDMRs exhibited reduced gene expression in cancer when compared to normal endometrium, whereas TFs associated with EAC ce-hypoDMRs exhibited increased expression. Similarly, in GBM, we observed specific incidences of methylation loss over regions bearing marks of active enhancers in alternative cell types, accompanying an increased expression of encompassed motif TFs. These results suggest that altered TF abundance may likely be driving the differential methylation patterns over regulatory regions in these cancers.

GO enrichment analyses of EAC ce-hyperDMR sets that contained clusters of enriched TF-binding motifs often enriched for GO terms associated with the original cell type—primarily uterine-specific disease terms. Although GBM ce-hyperDMRs did not show a similar enrichment of brain-specific disease terms, examination of their annotations across numerous normal brain and developing brain tissues revealed that hundreds more GBM hyperDMRs could be annotated as enhancers in adult brain tissues rather than in fetal brain. In addition, H3K4me1 and H3K27ac peaks within adult brain tissues were more commonly found in GBM hyperDMRs compared with peaks in developing brain tissues, suggesting that enhancers gaining methylation in GBM are more active in maintaining adult brain function as opposed to developing brain function. In contrast, ce-hypoDMRs with enriched TF-binding motifs were primarily associated with cancer-related GO terms. These results are consistent with the “cancer cell-identity crisis” hypothesis^67^: during carcinogenesis, tissue-specific enhancers may become methylated and silenced in addition to the silencing of tissue-specific TFs, contributing to the loss of original cellular identity. Hypomethylated cancer enhancers and associated upregulated TFs may also contribute to carcinogenesis by activating pro-growth, pro-migration pathways, and genes specific to other cell types, resulting in a deregulated cell fate. This concept is further illustrated by two examples of loss of methylation in GBM over enhancers active in a distant tissue type accompanied by increased expression of the predicted TF binding the enhancer and the target gene (Fig. 3d, e).

As TEs have been shown to harbor regulatory elements and have been routinely filtered out in methylation array-based studies, we examined the methylation status across various TE subfamilies in both cancer types. A large proportion of both EAC and GBM hyper- and hypoDMR-overlapped TEs had either enhancer and/or promoter potential, as determined by Roadmap’s reference human epigenome annotations. Distinct cancer-specific methylation abnormalities were found in GBM and EAC, possibly associated with tissue-specific activity, consistent with the observation that TEs can play tissue-specific enhancer roles^68^. However, enrichment of the retrotransposon subfamily MER52A was observed in both cancer-type hypoDMRs and across several additional cancer types, suggesting a potential role for this subfamily in carcinogenesis. Finally, several instances of hypomethylated TEs exhibited the active promoter histone mark, H3K4me3, in the U87 cell line (GBM), suggesting that a shared cancer mechanism may include altering the gene regulatory landscape through the demethylation of regulatory elements harbored within TEs.

## Methods

### Statistics and reproducibility

A description of all statistical methods used for each test can be found in the specific sub-sections below.

### DMR calling guidelines

For a genomic region to be called an EAC DMR, the region must have been differentially methylated between the cancer and the normal endometrium in at least two of the three EAC samples^25^. For a genomic region to be called a GBM DMR, the region must have been differentially methylated between the cancer and both normal brain samples in at least two of the five GBM samples^26^. In both cases, DMRs were defined at a 500 base pair resolution using the M&M tool^41^. The M&M tool integrates MeDIP-seq and MRE-seq data from two different samples and determines regions where the methylation levels are significantly different. In both previous studies where DMRs were called^25,26^, default parameters were set, which included “mreratio = 3/7” (the ratio of the percentage of unmethylated genome to the percentage that is methylated), “method = ‘XXYY’” (specifying to use MeDIP and MRE in testing), “psd = 2” (pseudo count added to MeDIP and MRE reads), “mkadded = 1” (pseudo count added to the number of CpGs in total and MRE-CpGs), “a = 1e − 16” (*p*-value cutoff when sum of observations is smaller than “top”), “cut = 100” (*p*-value cutoff when less than the sum of observations), and “top = 500” (*p*-value cutoff when less than the sum of observations and *p*-values < “a”). Additional default parameters used for selecting significant DMRs included “up = 1.45” (minimum threshold for MeDIP1/MeDIP2 read ratio), “p.value.MM = 0.01” (*p*-value threshold), “p.value.SAGE = 0.01” (SAGE *p*-value threshold), “*q*-value = 0.00005” (*q*-value threshold), “cutoff = ‘*q*-value’” (measurement to use to call significance), and “quant = 0.6” (minimum threshold for the rank of the absolute value of the difference between MeDIP1 and MeDIP2).

### Determining significance of DMR overlap

To calculate the significance of hyperDMRs and hypoDMRs shared by EAC and GBM, a hypergeometric test was performed using the phyper() function in R. More specifically, for calculating the significance of shared hyperDMRs, the values considered were as follows: 10,178 (total GBM hyperDMRs), 18,278 (total EAC hyperDMRs), 2760 (shared hyperDMRs), and 5,196,471 (number of 500 bp genomic regions with at least 1 CpG, and MeDIP and/or MRE signal in at least 1 sample). To calculate the significance of observing at least 2760 shared hyperDMRs, we used “lower.tail=FALSE,” as well as subtracted 1 from our “*x*” value (2760 − >2759). For calculating the significance of shared hypoDMRs, the values considered were as follows: 4494 (total GBM hypoDMRs), 8712 (total EAC hypoDMRs), 195 (shared hypoDMRs), and 5,196,471 (number of 500 bp genomic regions with at least 1 CpG, and MeDIP and/or MRE signal in at least 1 sample). To calculate the significance of observing at least 195 shared DMRs, we used “lower.tail=FALSE,” and subtracted 1 from our “*x*” value (195 − >194). To calculate the expected number of shared hyper/hypoDMRs, the following equation was used:

1 $${\mathrm{Expected}}\, {\mathrm{No.}}\, {\mathrm{of}}\, {{\mathrm{shared}}\,{{\mathrm{DMRs}}}}=\left(\frac{\mathrm{No.}}\, {{{\mathrm{of}}\,{{\mathrm{EAC}}\,{{\mathrm{DMRs}}}}}}{\mathrm{No.}}\,{{{\mathrm{of}}\,{{\mathrm{background}}\,500\,{\mathrm{bp}}\,{\mathrm{bins}}}}}\right)\ast {\mathrm{No.}}\,{\mathrm{of}}\,{{\mathrm{GBM}}\,{{\mathrm{DMRs}}}}$$

### Methylation validation using TCGA array data

Hg19-aligned TCGA methylation array-based datasets were downloaded from https://gdc.cancer.gov using the gdc-client (v1.6.0) for the following cancers: adrenocortical carcinoma, bladder urothelial carcinoma, breast invasive carcinoma, cervical squamous cell carcinoma and endocervical adenocarcinoma, cholangiocarcinoma, colon adenocarcinoma, lymphoid neoplasm diffuse large B-cell lymphoma, esophageal carcinoma, GBM, head and neck squamous cell carcinoma, kidney chromophobe, kidney renal clear cell carcinoma, kidney renal papillary cell carcinoma, acute myeloid leukemia, brain lower grade glioma, liver hepatocellular carcinoma, lung adenocarcinoma, lung squamous cell carcinoma, mesothelioma, ovarian serous cystadenocarcinoma, pancreatic adenocarcinoma, pheochromocytoma and paraganglioma, prostate adenocarcinoma, rectum adenocarcinoma, sarcoma, skin cutaneous melanoma, stomach adenocarcinoma, testicular germ cell tumors, thyroid carcinoma, thymoma, UCEC, uterine carcinosarcoma, and uveal melanoma. Methylation files corresponding to the same patient ID were averaged at each probe location. The average methylation values over probes overlapping DMRs were calculated for each tumor sample and normal sample (when available).

### Genomic characterization of DMRs

To determine the genomic landscape of each DMR class, we considered the following genomic regions:

- Promoters (1 kb core (500 bp upstream to 500 bp downstream the TSS) and 2.5 kb (2 kb upstream the TSS to 500 bp downstream the TSS), defined by refGene (last updated: 3 April 2016), downloaded from the UCSC Gene Annotation Database^69^ (http://hgdownload.soe.ucsc.edu/goldenPath/hg19/database/)).
- Unmasked CpG Islands (last updated: 1 June 2014), downloaded from the UCSC Gene Annotation Database^69^ (http://hgdownload.cse.ucsc.edu/goldenpath/hg19/database/).
- Gene bodies, defined by refGene (last updated: 3 April 2016), downloaded from the UCSC Gene Annotation Database^69^ (http://hgdownload.soe.ucsc.edu/goldenPath/hg19/database/)).
- Fantom 5 Enhancers, human permissive enhancers phase 1 and 2 (http://fantom.gsc.riken.jp/5/datafiles/latest/extra/Enhancers/)^42,43^.
- VISTA Enhancers (1745 human enhancers downloaded on 21 December 2015) human elements^44^ (Supplementary Data 5).
- Super enhancers (defined by dbSUPER^45^).
- Intergenic regions, defined by refGene (last updated: 3 April 2016), downloaded from the UCSC Gene Annotation Database^69^ (http://hgdownload.soe.ucsc.edu/goldenPath/hg19/database/)).

For each DMR class, we computed the fraction of DMR nucleotides that overlapped each genomic category. As these genomic categories are not mutually exclusive, DMR positions may have been counted more than once if they applied to multiple categories. Therefore, the percentages for each DMR group may sum to more than 1. Background regions considered when calculating enrichment were 500 kb genomic regions that excluded the ends of chromosomes, excluded bins overlapping blacklisted CpGs, excluded bins not containing at least one MeDIP and/or MRE read for at least one sample, excluded bins not containing at least one CpG, and excluded those on chrY (and chrX for GBM).

Enrichment was then calculated as:

2 $${\mathrm{Enrichment}}=\frac{ \% \,{\mathrm{of}}\,{\mathrm{DMR}}\,{\mathrm{nucleotides}}\,{\mathrm{overlapping}}\,{\mathrm{genomic}}\,{\mathrm{feature}}}{ \% \,{\mathrm{of}}\,{\mathrm{background}}\,{\mathrm{region}}\,{\mathrm{base}}\,{\mathrm{pairs}}\,{\mathrm{considered}}\,{\mathrm{overlapping}}\,{\mathrm{genomic}}\,{\mathrm{feature}}}$$

### Epigenetic characterization of DMRs

To determine the distribution of epigenomic annotations for each DMR group, we used chromHMM maps predefined for 80 cell and tissue types^32^, downloaded from Roadmap Epigenomics Data Portal, https://egg2.wustl.edu/roadmap/web_portal/ (Supplementary Data 5). For each DMR group, we calculated the percentage of DMR bps that overlapped each feature (defined according to the 18-state chromHMM model) in each cell/tissue type. To calculate enrichment, we divided the percentage of DMR bps overlapping the feature in the cell/tissue type by the percentage of background bps overlapping the feature in the cell/tissue type. Genomic regions considered for background purposes were defined as described above in “Genomic characterization of DMRs”.

### Polycomb binding enrichment within DMRs

To determine whether GBM hyperDMRs were enriched for polycomb binding in normal brain, we first downloaded control-normalized EZH2 ChIP-seq data for ENCODE’s NH-A sample (GSM1003532) in BigWig format and converted the file to bed format^47^. We then calculated the average normalized signal in GBM-unique hyperDMRs and GBM/EAC shared hyperDMRs, as well as the average normalized signal in background regions (described above in “Genomic characterization of DMRs”). Enrichment was then calculated as the ratio of the average signal in the DMR group to the average signal in the genomic background.

### DMR overlap to 450k and 850k array probes

If a DMR contained at least one probe found on the 450k array or 850k array, the DMR was considered identifiable via the 450k platform or 850k platform, respectively. Locations of Illumina HumanMethylation450 BeadChip probes (Infinium HumanMethylation450K v1.2) were downloaded from https://support.illumina.com/array/array_kits/infinium_humanmethylation450_beadchip_kit/downloads.html (“HumanMethylation450 v1.2 Manifest File (CSV Format)”). Locations of Illumina MethylationEPIC BeadChip probes (850K array) were downloaded from https://support.illumina.com/array/array_kits/infinium-methylationepic-beadchip-kit/downloads.html (“Infinium MethylationEPIC v1.0 B4 Manifest File (CSV Format)”).

### DMR overlap with potential regulatory regions

We identified regions of the genome with possible regulatory function as any 200 bp region that was annotated as one of the following chromatin states in at least one of the cell or tissue types listed above: 1_TssA (Active TSS), 2_TssFlnk (Flanking Active TSS), 3_TssFlnkU (Flanking TSS Upstream), 4_TssFlnkD (Flanking TSS Downstream), 7_EnhG1 (Genic Enhancers 1), 8_EnhG2 (Genic Enhancers 2), 9_EnhA1 (Active Enhancers 1), 10_EnhA2 (Active Enhancers 2), and 11_EnhWk (Weak enhancers). We then calculated the percentage of DMR base pairs that overlapped regions of regulatory potential. To calculate enrichment for EAC DMRs, the background was calculated as the percentage of hg19 base pairs with chromHMM 18-state annotations for the cell and tissue types above, excluding chrY and chrM, those that overlapped blacklisted CpG 500 bp bins, bins without a CpG, and bins without MeDIP and/or MRE signal in at least one sample (2,170,900,000 bp) that met the above criteria (1,109,392,000 bp (51.10%)). The background GBM was calculated similarly, additionally excluding chrX (of 2,076,745,800 bp, 1,081,211,800 bp (52.06%) met the above criteria).

We then calculated the percentage of DMR bps that overlapped specific types of regulatory regions: promoters (defined according to refGene and Roadmap chromHMM 18-state reference epigenomes), active enhancers (defined according to Roadmap chromHMM 18-state reference epigenomes), and both promoters and active enhancers. Regions of active enhancer potential were defined as any region annotated as “9_EnhA1” or “10_EnhA2” in at least 1 of the 80 tissues or cell-type chromHMM 18-state models described above. Promoter regions were defined as any region annotated as “1_TssA,” “2_TssFlnk,” “3_TssFlnkU,” or “4_TssFlnkD” in at least 1 of the 80 tissues or cell-type chromHMM 18-state models in addition to regions defined using refGene TSS annotations, where a promoter spanned 2 kb upstream the TSS to 500 bp downstream the TSS.

### TSG core promoter DMR overlap analysis

A human TSG list containing 1217 genes was obtained from the TSGene Tumor Suppressor Gene Database^70,71^ (http://bioinfo.mc.vanderbilt.edu/TSGene/Human_TSGs.txt); however, only 1216 of these genes could be identified with RefSeq (missing gene: TRP53COR), so we proceeded with the list of 1216 TSGs. The TSS of each TSG was identified using RefSeq and then TSG core promoter regions were defined as the region spanning 500 bp upstream to 500 bp downstream the TSS. All transcripts for each TSG were considered.

### Assigning enhancer regions to genes

A list of all genomic positions that were annotated as either state “9_EnhA1” or “10_EnhA2” in at least 1 Roadmap reference epigenomes listed above, based on the chromHMM 18-state model^32^, was compiled. DMRs not overlapping core promoter regions (1 kb regions centered around TSSs) were overlapped to these enhancer-potential regions. DMRs overlapping enhancers were assigned to the gene with the nearest TSS. If the nearest TSS was >500,000 bp away, the DMR was not assigned to any gene.

### Gene expression changes associated with TSG DNA methylation alterations

Normalized TCGA RNA-seq data (level-3, reads per kilobase of transcript, per million mapped reads (RPKM)) and clinical metadata of EAC, their matched-control samples, and GBM were downloaded from the Genomic Data Commons Data Portal (https://portal.gdc.cancer.gov/). Expression of normal brain samples (*n* = 28, frontal cortex) was downloaded from GTEx. TSGs with core promoters and/or active enhancers (assigned as described above) overlapping EAC hyperDMRs and/or GBM hyperDMRs were identified. This resulted in a list of 350 TSGs with an EAC hyperDMR overlapping the core promoter and/or an active enhancer, and 245 TSGs with a GBM hyperDMR overlapping the core promoter and/or an active enhancer. However, of the 350 and 245 TSGs, only 310 and 210 had available expression data (see Supplementary Data 3). In the case of EAC hyperDMRs, two TSGs with regulatory regions overlapping DMRs (BRINP1 and CCAR2) had the same alias: DBC1. As the expression data were associated with the alias, DBC1 was only counted once. For each TSGs with available RNA-seq data, the mean RPKM value was calculated for both cancer (EAC or GBM) and normal (normal endometrium or normal brain), and the expression fold change was calculated as:

3 $${{{{\rm{TSG}}}\, {\mathrm{expression}}\, {\mathrm{fold}}\, {\mathrm{change}}}}={{\log}}2\left(\frac{{{{\rm{TSG}}}\, {\mathrm{mean}}\, {\mathrm{RPKM}}\, {\mathrm{in}}\, {\mathrm{cancer}}}}{{{{\rm{TSG}}}\, {\mathrm{mean}}\,{\mathrm{RPKM}}\, {\mathrm{in}}\, {\mathrm{normal}}}}\right)$$

### GO enrichment for merged ceDMRs

Consecutive 500 bp DMRs were merged for each DMR group (EAC hyperDMRs, GBM hyperDMRs, EAC hypoDMRs, and GBM hypoDMRs) and any merged DMRs that overlapped promoter regions (refGene annotations, 2.5 kb) were discarded. The remaining DMRs were filtered to only include those that overlapped a region annotated as an active enhancer (chromHMM 18-states “9_EnhA1” or “10_EnhA2”) in at least 1 of the 80 tissues/cell types mentioned above. GREAT^72^ was then run with the remaining DMRs (each group separately), using version 3.0.0 and default parameters (including the whole genome as background). GO biological processes terms that were significant in both the binomial and hypergeometric tests (BinomFDRQ ≤ 0.05 and HyperFDRQ ≤ 0.05), were within the top 500 ranked Binomial test terms and had a region fold enrichment ≥ 2 were considered.

### Apoptosis pathway genes with DMRs overlapping promoters and enhancers

Apoptosis-associated genes were obtained from KEGG^73^ (Entry: hsa04210, *n* = 140). Genes with a DMR overlapping their promoter (refGene, 2.5 kb) were identified. A list of active enhancer locations (chromHMM 18-states “9_EnhA1” and “10_EnhA2” in at least 1 of the 80 tissue/cell types listed above) was obtained and active enhancers (unmerged) that overlapped promoters (refGene, 2.5 kb) were removed. Remaining active enhancer regions were then assigned to the nearest gene (shortest distance to the TSS) and those that were assigned to apoptosis genes and that overlapped DMRs were identified.

### TF expression differences in EAC vs. normal endometrium

To determine whether there was a correlation between ceDMRs and changes in TF expression, we used publicly available RNA-seq data from TCGA (https://portal.gdc.cancer.gov/). TFs with enriched motifs (see method below in “Motif and GO analysis for ceDMRs” with the exception: *q*-value ≤ 0.01) present in at least 20% of the DMRs were considered. Genes with an RPKM value ≤ 1 were excluded. RPKM values were then log2 transformed and *z*-scored.

### Motif and GO analysis for ceDMRs

#### EAC ce-hyperDMRs

The Homer^58^ (v4.9) function “findMotifsGenome.pl” was run using as input EAC ce-hyperDMRs: EAC hyperDMRs that did not overlap RefSeq promoters but did overlap an active enhancer annotation (“9_EnhA1” or “10_EnhA2”) in at least 1 of 80 tissues/cell types from Roadmap’s chromHMM 18-state model predictions (listed above). EAC ce-hypoDMRs were used as background. Aside from the pre-specified background and the flag “-size given,” default parameters were used to detect enriched TFBSs using known vertebrate motifs (*n* = 364). Resulting enriched motifs were then filtered to include only those with a *q*-value ≤ 0.05. Remaining motifs were then matched with their most likely TF using Homer’s Motif database and the expression of each TF was calculated in TCGA EAC samples and normal endometrium samples. TFs with a significant loss of expression in the EAC samples relative to the normal samples (*t*-test, Benjamini-Hochberg corrected) were retained. The Homer2 function “annotatePeaks.pl” was then run to identify the location of each enriched motif within the EAC ceDMRs, using default parameters. The binary matrix of EAC ce-hyperDMRs and enriched motifs (where 1 indicated the presence of the motif in the DMR and 0 indicated the absence) was then clustered using R’s heatmap.2 function with distance method “euclidean” and clustering method “complete.” To identify clusters of TFs, the dendrogram was cut at a height of 42. All resulting groups that had more than one TF were called a cluster. For each cluster, DMRs that contained an enriched TF-binding motif were then checked for enriched Disease Ontology terms using GREAT^72^ v3.0 and parameters: Species Assembly: Human GRCh37, Background regions: Whole genome; and default association rule settings. The top five enriched Disease Ontology terms are displayed for each cluster.

#### GBM ce-hyperDMRs

The same methods described for EAC ce-hyperDMRs were used here, with the following exceptions. GBM ce-hyperDMRs: GBM hyperDMRs that did not overlap RefSeq promoters but did overlap an active enhancer annotation (“9_EnhA1” or “10_EnhA2”) in at least 1 of 80 tissues/cell types from Roadmap’s chromHMM 18-state model predictions (Supplementary Data 5) were used as input to the Homer2 (v4.9) function “findMotifsGenome.pl.” GBM ce-hypoDMRs were used as background. TFs with a significant loss of expression in TCGA GBM samples relative to normal brain were retained. To identify clusters, the dendrogram was cut at a height of 25.

#### EAC ce-hypoDMRs

The same methods described above were used here, with the following exceptions. EAC ce-hypoDMRs: EAC hypoDMRs that did not overlap RefSeq promoters but did overlap an active enhancer annotation (“9_EnhA1” or “10_EnhA2”) in at least 1 of 80 tissues/cell types from Roadmap’s chromHMM 18-state model predictions (listed above) were used as input to the Homer2 (v4.9) function “findMotifsGenome.pl.” EAC ce-hyperDMRs were used as background. TFs with a significant gain of expression in TCGA EAC samples relative to normal endometrium were retained. To identify clusters, the dendrogram was cut at a height of 30.

#### GBM ce-hypoDMRs

The same methods described above were used here, with the following exceptions. GBM ce-hypoDMRs: GBM hypoDMRs that did not overlap RefSeq promoters but did overlap an active enhancer annotation (“9_EnhA1” or “10_EnhA2”) in at least 1 of 80 tissues/cell types from Roadmap’s chromHMM 18-state model predictions (listed above) were used as input to the Homer2 (v4.9) function “findMotifsGenome.pl.” GBM ce-hyperDMRs were used as background. TFs with a significant gain of expression in TCGA GBM samples relative to normal brain were retained. To identify clusters, the dendrogram was cut at a height of 22.

### Data sources for enhancer hypomethylation examples

ChIA-PET HeLa RNAPol2: ENCODE data portal (https://www.encodeproject.org/). Adipose H3K4me1, H3K4me3, H3K27ac: processed adipose ChIP-seq data (bigWig: H3K4me1, H3K4me3, and H3K27ac) were downloaded from the ENCODE data portal (https://www.encodeproject.org/). A549 TCF12 ChIP-seq: ENCODE data portal (https://www.encodeproject.org/). ChIP-PET K562 RNAPol2: ENCODE data portal (https://www.encodeproject.org/). Adipose (subcutaneous, visceral) and brain (frontal cortex) RNA-seq: GTEx. GBM expression: TCGA (https://portal.gdc.cancer.gov/).

### Determining GBM hyperDMR overlap to H3K27ac and H3K4me1 peaks in adult and fetal brain samples

We began by starting with all 500 bp GBM hyperDMRs that overlapped state “7_Enh” (chromHMM 15-state model) in at least one fetal or adult Roadmap brain sample (E053, E054, E067, E068, E069, E070, E071, E072, E073, E074, E081, E082, and E125), and that did not overlap with refGene-defined 2.5 kb promoters. We then obtained H3K4me1 peak files from Roadmap (downloaded from Roadmap Epigenomics Data Portal (https://egg2.wustl.edu/roadmap/web_portal/)) for the following samples: E053, E054, E070, E081, E082, E125 (fetal brain) and E067, E068, E069, E071, E072, E073, E074 (adult brain), as well as H3K27ac narrow peak files from Roadmap (downloaded from Roadmap Epigenomics Data Portal (https://egg2.wustl.edu/roadmap/web_portal/)) for the following samples: E125 (fetal brain) and E067, E068, E069, E071, E072, E073, and E074 (adult brain). All H3K4me1 adult brain peak files were merged to generate one adult brain H3K4me1 signal file and all H3K4me1 fetal brain peak files were merged to generate one fetal brain H3K4me1 signal file. All H3K27ac adult brain peak files were merged to generate one adult brain H3K27ac signal file and the E125 H3K27ac peak file was used as the fetal brain H3K27ac signal file.

### TE DMR overlap and subfamily enrichment

RepeatMasker annotations were downloaded from the UCSC Genome Browser^69^. Simple repeats and low-complexity repeats were removed from annotation. The number of DMRs that overlapped TEs were determined using bedtools (v2.27.1). Regions of DMRs that overlapped TEs were extracted and the percentages of bps that overlapped genic promoters (RefSeq annotations, 2.5 kb) were determined. Regions not overlapping genic promoter annotations were then tested to see if they overlapped epigenomically defined promoter states (regions annotated as “1_TssA,” “2_TssFlnk,” “3_TssFlnkU,” or “4_TssFlnkD” in at least 1 of the 80 tissues or cell-type chromHMM 18-state models). Finally, remaining regions were tested to see if they overlapped active enhancer regions (region annotated as “9_EnhA1” or “10_EnhA2” in at least 1 of the 80 tissues or cell-type chromHMM 18-state models described above).

Subfamily enrichment was calculated as:

4w $${E}_{\mathrm{s}}=\frac{\frac{{n}_{\mathrm{TE}}}{{n}_{\mathrm{DMR}}}}{\frac{{N}_{\mathrm{TE}}}{{N}_{\mathrm{all}}}}$$

here *n*_TE_ is the number of DMRs containing TEs, *n*_DMR_ is the total number of DMRs, *N*_TE_ is the number of genomic windows overlapped with TEs in the human genome, and *N*_all_ is the number of 500 bp windows in the human genome (hg19).

### MER52A subfamily DNA methylation measurements across various normal and cancer tissues and cell types

WGBS data were downloaded from the Roadmap data portal (http://www.roadmapepigenomics.org/) and the ENCODE data portal (https://www.encodeproject.org/). The CpG sites were filtered to only include those that had at least 5× coverage. The average methylation level of each MER52A copy was calculated for generating boxplots and heat maps.

### H3K4me3 signal over hypomethylated TEs in GBM

H3K4me3 ChIP-seq signal (bigWig) file for U87 (normalized reads per million) was downloaded from GEO (GSM2634761). The bigWig file was visualized on the WashU Epigenome Browser^74,75^.

### Reporting summary

Further information on research design is available in the Nature Research Reporting Summary linked to this article.

## Supplementary information

Supplementary Information

Descriptions of Additional Supplementary Files

Supplementary Data 1

Supplementary Data 2

Supplementary Data 3

Supplementary Data 4

Supplementary Data 5

Reporting Summary

## Data Availability

The data analyzed in the present study can be accessed as described below. Source data used to generate manuscript figures are available in the Github repository: https://github.com/jaflynn5/EAC_GBM_comparative_epigenomics, which is also linked to the Zenodo repository with the identifier [DOI: 10.5281/zenodo.4637753]^76^. Any additional source data can be obtained from the corresponding authors upon reasonable request. EAC MeDIP-seq + MRE-seq: [GEO: GSE51565]. GBM MeDIP-seq + MRE-seq: [EGA: EGAS00001000685]. Hg19 RefSeq annotations (last updated: 3 April 2016), downloaded from the UCSC Gene Annotation Database (http://hgdownload.soe.ucsc.edu/goldenPath/hg19/database/)). Hg19 unmasked CpG islands (last updated: 1 June 2014), downloaded from the UCSC Gene Annotation Database (http://hgdownload.cse.ucsc.edu/goldenpath/hg19/database/). Fantom 5 Enhancers, human permissive enhancers phase 1 and 2: http://fantom.gsc.riken.jp/5/datafiles/latest/extra/Enhancers/. VISTA Enhancers (1745 human enhancers downloaded on 21 December 2015) human elements: https://enhancer.lbl.gov/. Super enhancers: https://asntech.org/dbsuper/. Blacklisted CpGs: http://genome.ucsc.edu/cgi-bin/hgFileUi?db=hg19&g=wgEncodeMapability. chromHMM 18-state maps and 15-state maps: downloaded from Roadmap Epigenomics Data Portal, https://egg2.wustl.edu/roadmap/web_portal/. NH-A EZH2 CHIP-seq: [GEO: GSM1003532]. 450k and 850k array probe locations: locations of Illumina HumanMethylation450 BeadChip probes (Infinium HumanMethylation450K v1.2) were downloaded from https://support.illumina.com/array/array_kits/infinium_humanmethylation450_beadchip_kit/downloads.html (“HumanMethylation450 v1.2 Manifest File (CSV Format)”). Locations of Illumina MethylationEPIC BeadChip probes (850K array) were downloaded from https://support.illumina.com/array/array_kits/infinium-methylationepic-beadchip-kit/downloads.html (“Infinium MethylationEPIC v1.0 B4 Manifest File (CSV Format)”). Tumor suppressor genes: https://bioinfo.uth.edu/TSGene/. TCGA EAC, normal endometrium, and GBM RNA-seq data (level-3, RPKM) and clinical metadata of EAC, GBM, and their matched-control samples were downloaded from the Genomic Data Commons Data Portal (https://portal.gdc.cancer.gov/). Adipose (subcutaneous, visceral) and brain (frontal cortex) RNA-seq: GTEx. Apoptosis gene list: KEGG^73^ (Entry: hsa04210, *n* = 140). ChIA-PET HeLa RNAPol2: ENCODE data portal (https://www.encodeproject.org/). Processed adipose ChIP-seq data (bigWig, H3K4me1, H3K4me3, and H3K27ac) were downloaded from the ENCODE data portal (https://www.encodeproject.org/). A549 TCF12 ChIP: ENCODE data portal (https://www.encodeproject.org/). ChIP-PET K562 RNAPol2: ENCODE data portal (https://www.encodeproject.org/). H3K27ac and H3K4me1 Peaks in Adult and Fetal Brain Samples: we obtained H3K4me1 peak files from Roadmap (downloaded from Roadmap Epigenomics Data Portal (https://egg2.wustl.edu/roadmap/web_portal/)) for the following samples: E053, E054, E070, E081, E082, E125 (fetal brain) and E067, E068, E069, E071, E072, E073, E074 (adult brain), as well as H3K27ac narrow peak files from Roadmap (downloaded from Roadmap Epigenomics Data Portal (https://egg2.wustl.edu/roadmap/web_portal/)) for the following samples: E125 (fetal brain) and E067, E068, E069, E071, E072, E073, E074 (adult brain). RepeatMasker annotations were downloaded from the UCSC Genome Browser^69^ https://hgdownload.soe.ucec.edu/download.html. WGBS data (thymus, ovary, pancreas, lung, mid-frontal cortex, brain germinal matrix, frontal cortex neuron, frontal cortex glia, atrium, sigmoid colon, colon tumor, colorectal cell line HCT116, breast myoepithelial, breast cancer HCC1954, liver, HepG2) were downloaded from the Roadmap data portal (http://www.roadmapepigenomics.org/) and the ENCODE data portal (https://www.encodeproject.org/). U87 H3K4me3 ChIP-seq [GEO: GSM2634761]. TCGA Infinium 450k array probe data for all available cancer types: https://portal.gdc.cancer.gov/. Normal glia RNA-seq: [GEO: GSE41826]. TCGA methylation array data: https://gdc.cancer.gov/.

## Code availability

Custom scripts generated for use in this study are available in the Github repository https://github.com/jaflynn5/EAC_GBM_comparative_epigenomics, which is also linked to the Zenodo repository with the identifier [DOI: 10.5281/zenodo.4637753]^76^. Any additional scripts can be obtained from the corresponding authors upon reasonable request. Software utilized include: R (v3.3.0), Homer2 (v4.9), and GREAT (v3.0).
