# Supplementary material for: Common DNA methylation dynamics in endometriod adenocarcinoma and glioblastoma suggest universal epigenomic alterations in tumorigenesis
Source: Commun Biol. 2021 May 21;4:607. doi: 10.1038/s42003-021-02094-1 (PMC8140130; doi:10.1038/s42003-021-02094-1)
Supplement: Supplementary file 1 — Supplementary Information [file 42003_2021_2094_MOESM1_ESM.pdf]

Supplementary Figures 1-5, Supplementary Tables 1 and 2, and Supplementary References

Common DNA methylation dynamics in endometriod adenocarcinoma and glioblastoma suggest universal epigenomic alterations in tumorigenesis

Jennifer A Karlow[1], Benpeng Miao[1,2], Xiaoyun Xing[1], Ting Wang[1,*], Bo Zhang[1,2,*]

* These authors jointly supervised this work.
Co-corresponding author emails: twang@wustl.edu and bzhang29@wustl.edu

[1]The Edison Family Center for Genome Sciences and Systems Biology, Department of Genetics, McDonnell Genome Institute, Washington University School of Medicine, 4515 McKinley Avenue, Campus Box 8510, St. Louis, MO 63110, USA
[2]Center of Regenerative Medicine, Department of Developmental Biology, Washington University School of Medicine, 4515 McKinley Avenue, St. Louis, MO 63110, USA

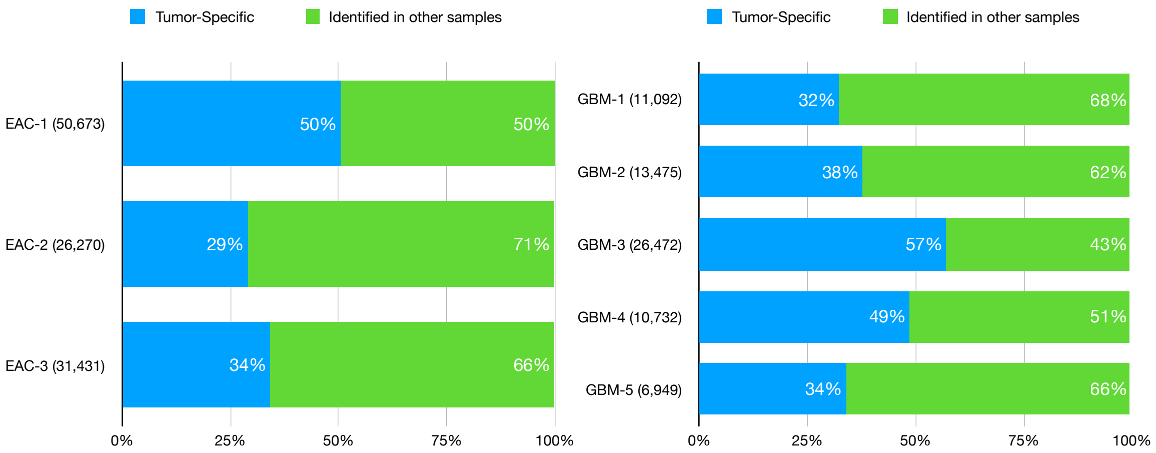

Supplementary Figure 1: DMR variability across tumors within cancer types. Number of DMRs identified for each tumor/normal comparison are depicted in parentheses adjacent the tumor ID. The percentage of each tumor's DMRs that were identified in at least one other tumor/normal comparison of the same cancer type are depicted in green ("Identified in other samples"). The percentage of each tumor's DMRs that were only identified in that tumor/normal pair for the particular cancer type are depicted in blue ("Tumor-Specific"). Left: EAC samples; Right: GBM samples.

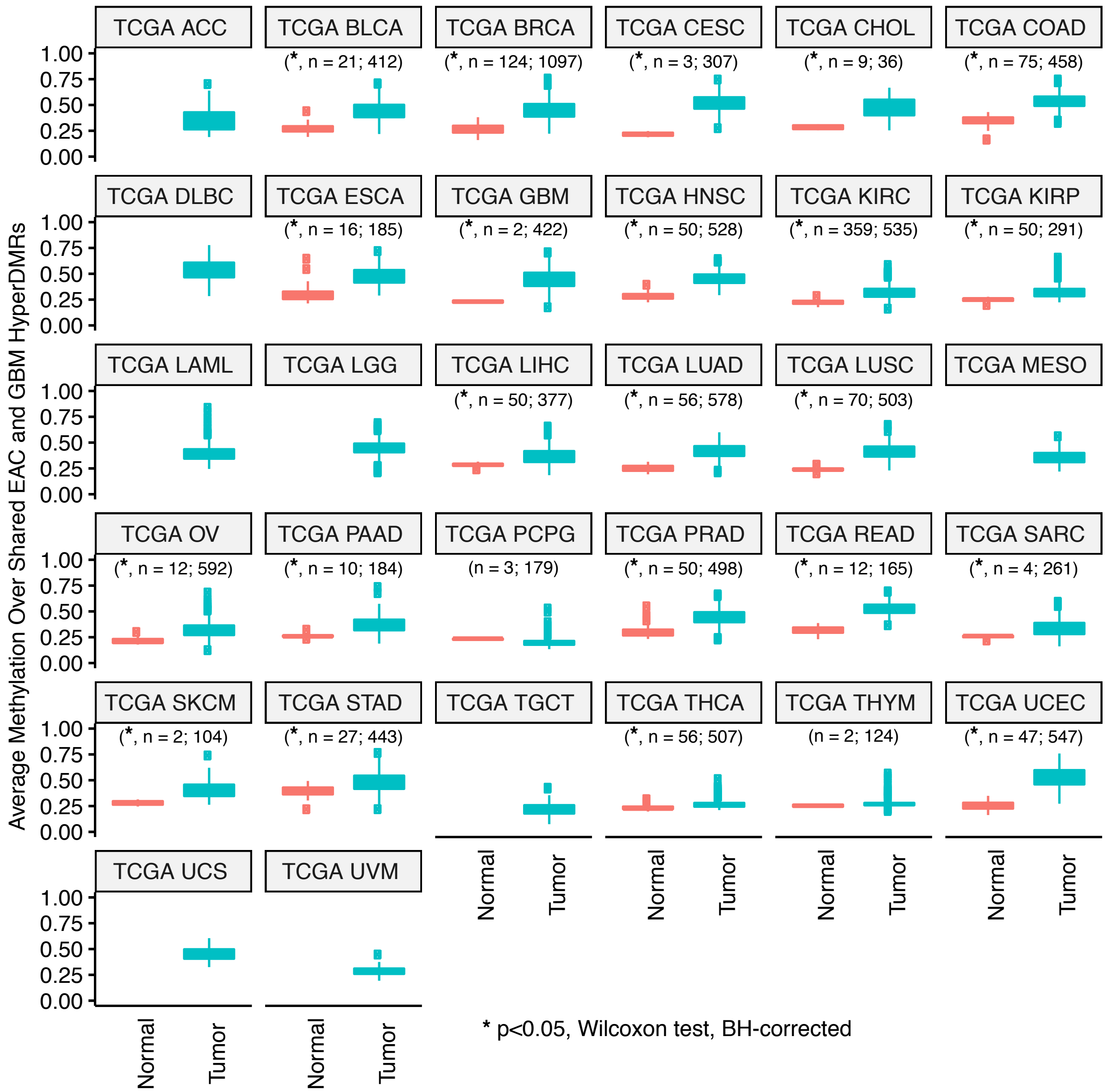

Supplementary Figure 2: Methylation Levels within TCGA Samples Over Shared EAC and GBM hyperDMRs. Average methylation over TCGA CpG probes for 32 cancer types, spanning shared EAC and GBM hyperDMRs in normal (red) and tumor (blue) samples, where data are available (n=number of normal cases; number of cancer type cases). Significant methylation changes (all in the form of methylation gain) were identified in 22/24 TCGA cancer types (t-tests, p<0.05, BH corrected).

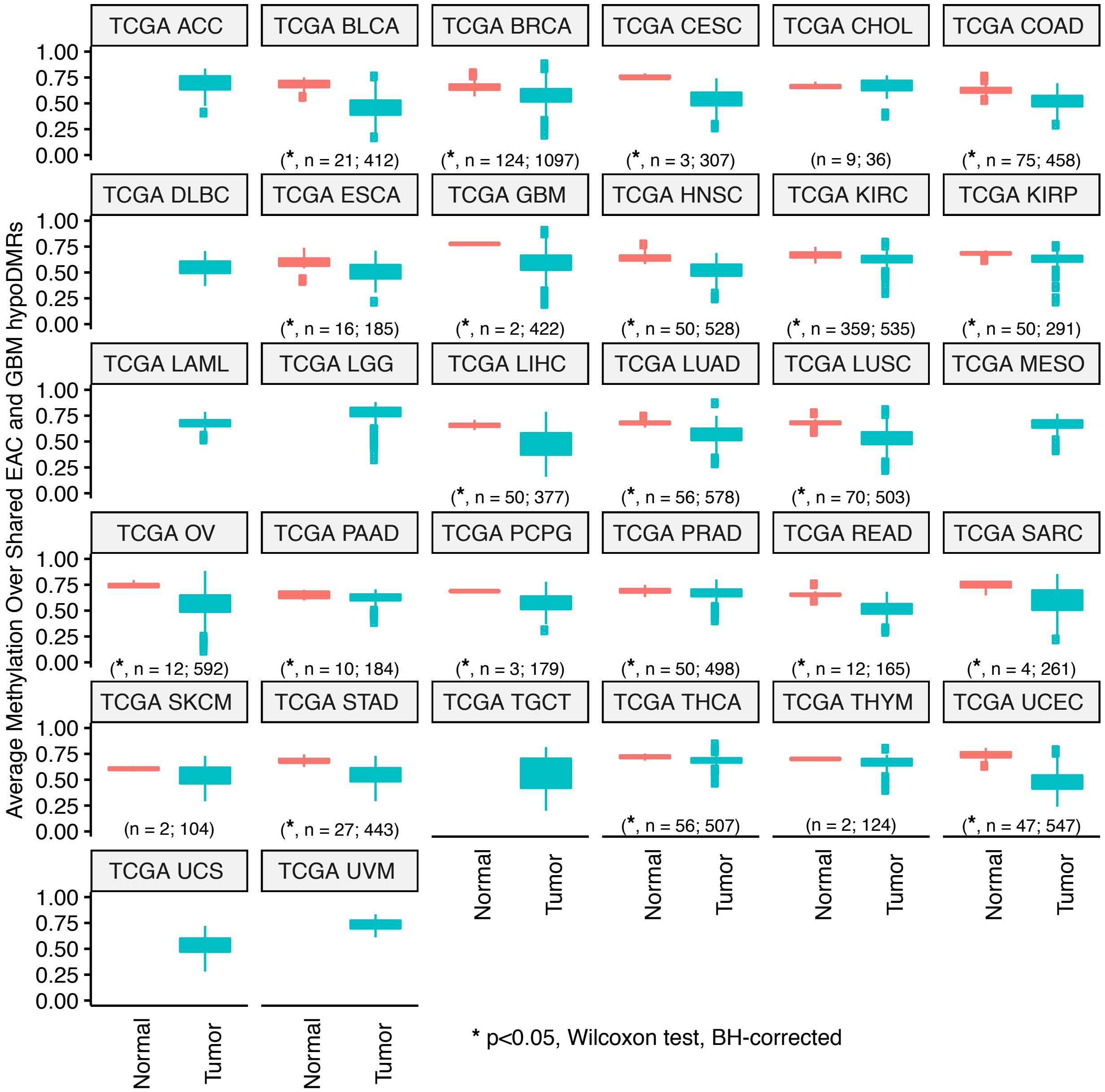

Supplementary Figure 3: Methylation Levels within TCGA Samples Over Shared EAC and GBM hypoDMRs. Average methylation over TCGA CpG probes for 32 cancer types, spanning shared EAC and GBM hypoDMRs in normal (red) and tumor (blue) samples, where data are available (n=number of normal cases; number of cancer type cases). Significant methylation changes (all in the form of methylation loss) were identified in 21/24 TCGA cancer types (t-tests, p<0.05, BH corrected).

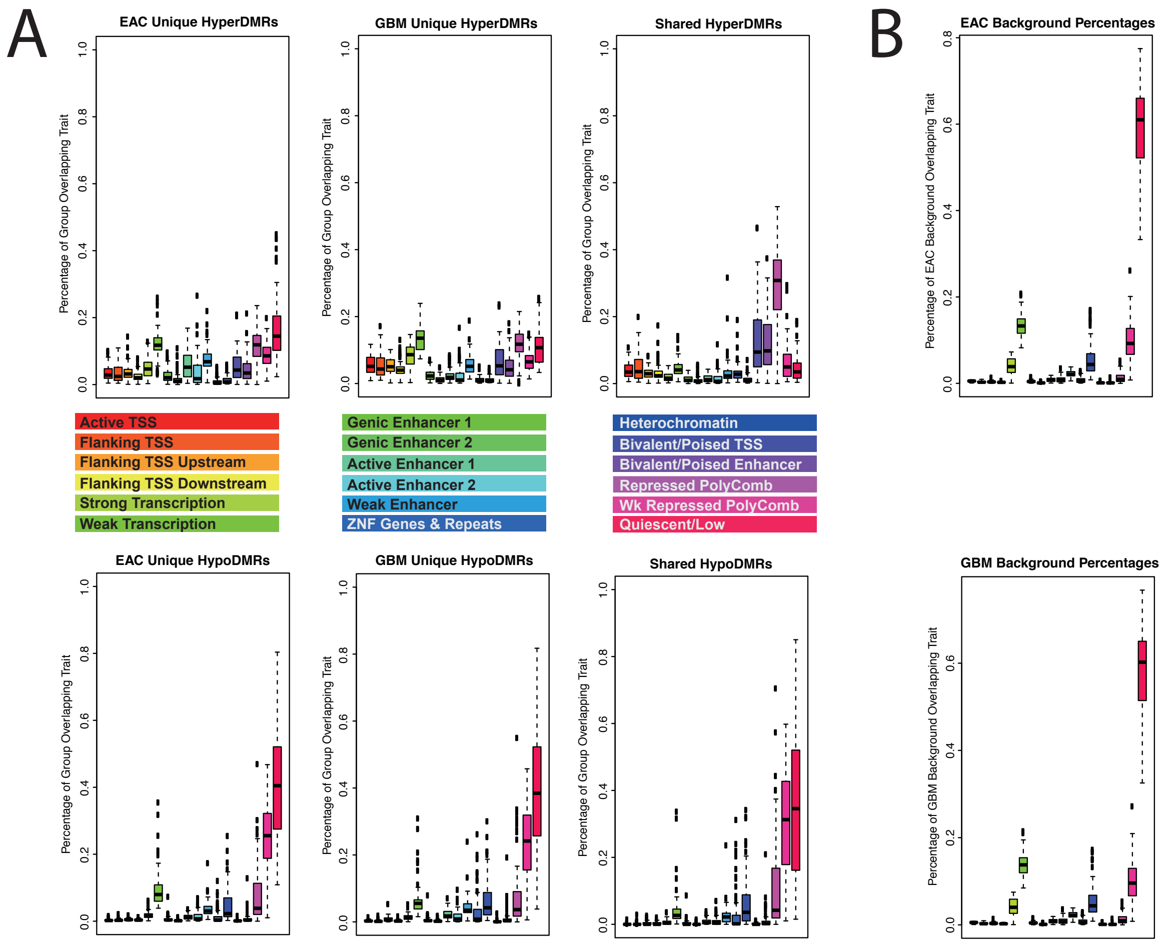

Supplementary Figure 4: Comparative Analysis of EAC and GBM DNA Methylation Abnormalities. EAC and GBM DMR (**A**) and genomic background (**B**) percentage overlaps with epigenetic annotations (chromHMM 18-states) across a variety of cell and tissue types (n=80, Human Roadmap Epigenome[1], see **Supplementary Data 5** for a complete list).

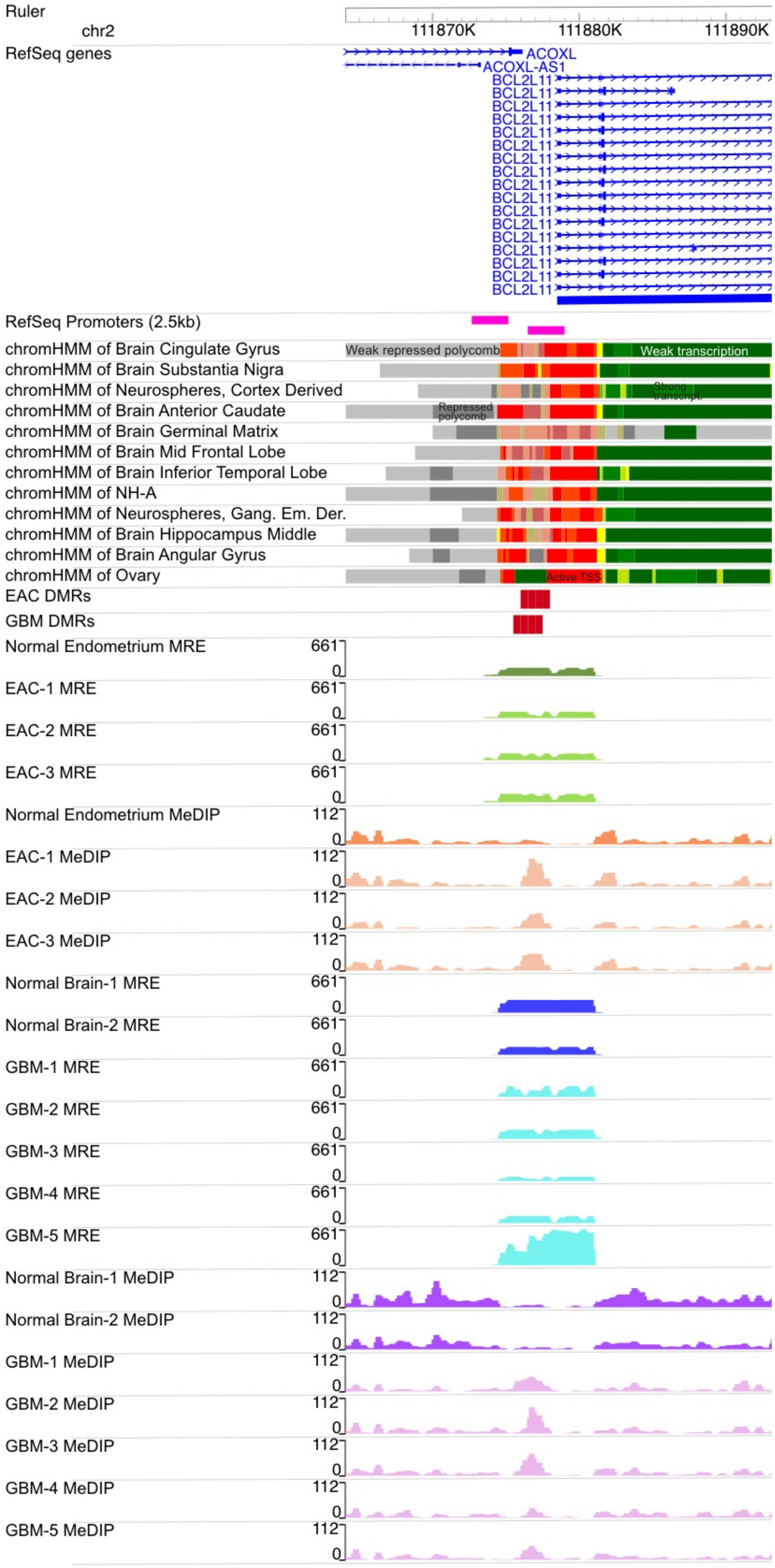
Ruler
chr2
111870K
111880K
111890K
RefSeq genes
ACOXL
ACOXL-AS1
BCL2L11
RefSeq Promoters (2.5kb)
chromHMM of Brain Cingulate Gyrus
Weak repressed polycomb
Weak transcription
chromHMM of Brain Substantia Nigra
chromHMM of Neurospheres, Cortex Derived
Strong transcript.
chromHMM of Brain Anterior Caudate
Repressed polycomb
chromHMM of Brain Germinal Matrix
chromHMM of Brain Mid Frontal Lobe
chromHMM of Brain Inferior Temporal Lobe
chromHMM of NH-A
chromHMM of Neurospheres, Gang. Em. Der.
chromHMM of Brain Hippocampus Middle
chromHMM of Brain Angular Gyrus
chromHMM of Ovary
Active TSS
EAC DMRs
GBM DMRs
Normal Endometrium MRE
EAC-1 MRE
EAC-2 MRE
EAC-3 MRE
Normal Endometrium MeDIP
EAC-1 MeDIP
EAC-2 MeDIP
EAC-3 MeDIP
Normal Brain-1 MRE
Normal Brain-2 MRE
GBM-1 MRE
GBM-2 MRE
GBM-3 MRE
GBM-4 MRE
GBM-5 MRE
Normal Brain-1 MeDIP
Normal Brain-2 MeDIP
GBM-1 MeDIP
GBM-2 MeDIP
GBM-3 MeDIP
GBM-4 MeDIP
GBM-5 MeDIP
661
112
0

Supplementary Figure 5: Browser[2,3] view of EAC and GBM hyperDMRs overlapping *BCL2L11* regulatory regions

First panel displays RefSeq gene locations across genomic coordinates (shown in ruler at the top), focused in on the *BCL2L11* promoter and surrounding regions. The second panel displays the locations of RefSeq promoters, defined as the regions spanning 2kb upstream to 500bp downstream each transcription start site (TSS). The proceeding 12 tracks display predicted chromatin states for various cell and tissue types surrounding the *BCL2L11* promoter, provided by the Roadmap Epigenomics Consortium[1]. A full catalogue of color IDs has been made available[1]. The next 2 tracks display the locations of EAC and GBM DMRs, respectively (red = hyperDMR; blue = hypoDMR). The following track displays normal endometrium MRE-seq data, and the next 3 tracks display MRE-seq data across 3 EAC samples. Similarly, next is a track displaying normal endometrium MeDIP-seq data followed by MeDIP-seq data tracks for the same 3 EAC samples. Finally, in a similar manner, 2 normal brain MRE-seq tracks are followed by 5 GBM MRE-seq tracks, and 2 normal brain MeDIP-seq tracks are followed by 5 GBM MeDIP-seq tracks. (MeDIP-seq and MRE-seq tracks depict raw counts; “Gang. Em. Der.” = “Ganglion Eminence Derived”)

Supplementary Table 1: EZH2 binding enrichment within GBM hyperDMRs

	GBM-Unique HyperDMRs	Shared GBM/EAC HyperDMRs
Average EZH2 Signal Over DMRs	16.6406	35.8355
Average EZH2 Signal Over Background	3.51367	3.51367
Enrichment of EZH2 Signal in DMRs Over Background	4.73596	10.1989

ChIP-Seq data from ENCODE's NH-A sample (GSM1003532)[4]

Supplementary Table 2: Summary of DMRs containing CpG probes in the Infinium 450K and 850K platforms

	DMRs in cancer	Total	Infinium 450K		Infinium 850K	
			DMRs Covered	Percentage	DMRs Covered	Percentage
GBM	Hypermethylated	10178	7013	68.90%	7595	74.62%
	Hypomethylated	4494	829	18.45%	1483	33.00%
	Total	14672	7842	53.45%	9078	61.87%
EAC	Hypermethylated	18278	10706	58.57%	12423	67.97%
	Hypomethylated	8712	2102	24.13%	3272	37.56%
	Total	26990	12808	47.45%	15695	58.15%

Supplementary References

1. Roadmap Epigenomics Consortium, Kundaje A, Meuleman W, et al. Integrative analysis of 111 reference human epigenomes. Nature. 2015;518:317-329. doi:10.1038/nature14248
2. Zhou X, Maricque B, Xie M, et al. The human epigenome browser at Washington University. *Nat Methods*. 2011;8(12):989-990. doi:10.1038/nmeth.1772
3. Li D, Hsu S, Purushotham D, Sears RL, Wang T. WashU Epigenome Browser update 2019. *Nucleic Acids Res*. 2019;47(W1):W158-W165. doi:10.1093/nar/gkz348
4. Dunham I, Kundaje A, Aldred SF, et al. An integrated encyclopedia of DNA elements in the human genome. *Nature*. 2012;489(7414):57-74. doi:10.1038/nature11247
5. Haeussler M, Zweig AS, Tyner C, et al. The UCSC Genome Browser database: 2019 update. *Nucleic Acids Res*. 2019;47(D1):D853-D858. doi:10.1093/nar/gky1095
6. Zhao M, Sun J, Zhao Z. TSGene: A web resource for tumor suppressor genes. *Nucleic Acids Res*. 2013;41(D1):D970-976. doi:10.1093/nar/gks937
7. Zhao M, Kim P, Mitra R, Zhao J, Zhao Z. TSGene 2.0: An updated literature-based knowledgebase for Tumor Suppressor Genes. *Nucleic Acids Res*. 2016;44(D1):D1023-D1031. doi:10.1093/nar/gkv1268
8. McLean CY, Bristor D, Hiller M, et al. GREAT improves functional interpretation of cis-regulatory regions. *Nat Biotechnol*. 2010;28(5):495-501. doi:https://doi.org/10.1038/nbt.1630
9. Visel A, Minovitsky S, Dubchak I, Pennacchio LA. VISTA Enhancer Browser - A database of tissue-specific human enhancers. *Nucleic Acids Res*. 2007;35(suppl_1):D88-D92. doi:10.1093/nar/gkl822
