# Supplementary material for: Common DNA methylation dynamics in endometriod adenocarcinoma and glioblastoma suggest universal epigenomic alterations in tumorigenesis
Source: Commun Biol. 2021 May 21;4:607. doi: 10.1038/s42003-021-02094-1 (PMC8140130; doi:10.1038/s42003-021-02094-1)
Supplement: Supplementary file 2 — Descriptions of Additional Supplementary Files [file 42003_2021_2094_MOESM2_ESM.pdf]

Descriptions of Additional Supplementary Files

Supplementary Data 1
Description: Tumor suppressor gene DMR overlap analysis.

Supplementary Data 2
Description: Tumor suppressor genes with core promoter hyperDMRs in both EAC and GBM.

Supplementary Data 3
Description: Tumor suppressor gene promoter and/or enhancer overlaps with EAC and GBM hyperDMRs. The full list of 1217 tumor suppressor genes (TSGs) analyzed in this study[6,7] are displayed, along with any relevant aliases. A value of 1 is given in the 3rd and 4th column if the gene's promoter or enhancer, respectively, overlaps with the hyperDMR set (1st tab: EAC hyperDMR overlaps, 2nd tab: GBM hyperDMR overlaps). The union of all TSGs with either promoter and/or enhancer overlaps to the hyperDMR set is given to the right. In the case of EAC hyperDMRs, two TSGs with regulatory regions overlapping DMRs (*BRINP1* and *CCAR2*) had the same alias: *DBC1*. Since the expression data was associated with the alias, *BRINP1* and *CCAR2* were replaced by *DBC1* on the right. TSGs without expression data are highlighted in orange.

Supplementary Data 4
Description: GREAT[8] GO Biological Processes significantly enriched terms for DMRs overlapping active enhancers outside of promoters (ceDMRs). Terms reported are significant in both the binomial and hypergenometric tests (BinomFDRQ<=0.05 and HyperFDRQ<=0.05, are within the top 500 ranked binomial test terms, and have a region fold change of 2 or greater). Tab1: EAC ce-hyperDMRs, Tab2: EAC ce-hypoDMRs, Tab3: GBM ce-hyperDMRs, Tab4: ce-GBM hypoDMRs.

Supplementary Data 5
Description: VISTA Enhancer IDs[12] and Roadmap chromHMM 18-state map IDs[1].
