# Supplementary material for: Common DNA methylation dynamics in endometriod adenocarcinoma and glioblastoma suggest universal epigenomic alterations in tumorigenesis
Source: Commun Biol. 2021 May 21;4:607. doi: 10.1038/s42003-021-02094-1 (PMC8140130; doi:10.1038/s42003-021-02094-1)
Supplement: Supplementary file 8 — Reporting Summary [file 42003_2021_2094_MOESM8_ESM.pdf]

nature research

Corresponding author(s): Ting Wang and Bo Zhang

Last updated by author(s): Mar 23, 2021

Reporting Summary

Nature Research wishes to improve the reproducibility of the work that we publish. This form provides structure for consistency and transparency in reporting. For further information on Nature Research policies, see our Editorial Policies and the Editorial Policy Checklist.

Statistics

For all statistical analyses, confirm that the following items are present in the figure legend, table legend, main text, or Methods section.

n/a	Confirmed	
☐	☒	The exact sample size (*n*) for each experimental group/condition, given as a discrete number and unit of measurement
☐	☒	A statement on whether measurements were taken from distinct samples or whether the same sample was measured repeatedly
☐	☒	The statistical test(s) used AND whether they are one- or two-sided<br>*Only common tests should be described solely by name; describe more complex techniques in the Methods section.*
☒	☐	A description of all covariates tested
☐	☒	A description of any assumptions or corrections, such as tests of normality and adjustment for multiple comparisons
☐	☒	A full description of the statistical parameters including central tendency (e.g. means) or other basic estimates (e.g. regression coefficient) AND variation (e.g. standard deviation) or associated estimates of uncertainty (e.g. confidence intervals)
☐	☒	For null hypothesis testing, the test statistic (e.g. *F*, *t*, *r*) with confidence intervals, effect sizes, degrees of freedom and *P* value noted<br>*Give P values as exact values whenever suitable.*
☒	☐	For Bayesian analysis, information on the choice of priors and Markov chain Monte Carlo settings
☒	☐	For hierarchical and complex designs, identification of the appropriate level for tests and full reporting of outcomes
☒	☐	Estimates of effect sizes (e.g. Cohen's *d*, Pearson's *r*), indicating how they were calculated

Our web collection on statistics for biologists contains articles on many of the points above.

Software and code

Policy information about availability of computer code

Data collection	The gdc-client (v1.6.0) was used to download hg19-aligned TCGA methylation array-based datasets.
Data analysis	GREAT v3.0 was used to identify enriched ontology terms. Homer v4.9 was used to identify enriched motifs. R functions, including phyper() were used and indicated in the manuscript text. All custom code used in this manuscript has been deposited in and is available at the github repository: https://github.com/jaflynn5/EAC_GBM_comparative_epigenomics and in the Zenodo repository: DOI: 10.5281/zenodo.4637753.

For manuscripts utilizing custom algorithms or software that are central to the research but not yet described in published literature, software must be made available to editors and reviewers. We strongly encourage code deposition in a community repository (e.g. GitHub). See the Nature Research guidelines for submitting code & software for further information.

Data

Policy information about availability of data

All manuscripts must include a data availability statement. This statement should provide the following information, where applicable:

- Accession codes, unique identifiers, or web links for publicly available datasets
- A list of figures that have associated raw data
- A description of any restrictions on data availability

Data Availability:

The data analyzed in the present study can be accessed as described below.
• EAC MeDIP-seq + MRE-seq: [GEO: GSE51565]
• GBM MeDIP-seq + MRE-seq: [EGA: EGAS00001000685]
• Hg19 RefSeq annotations: (last updated: April 3, 2016), downloaded from the UCSC Gene Annotation Database (http://hgdownload.soe.ucsc.edu/goldenPath/

hg19/database/))
• Hg19 unmasked CpG islands: (last updated: June 1, 2014), downloaded from the UCSC Gene Annotation Database (http://hgdownload.cse.ucsc.edu/goldenpath/hg19/database/)
• Fantom 5 Enhancers, human permissive enhancers phase 1 and 2: (http://fantom.gsc.riken.jp/5/datafiles/latest/extra/Enhancers/)
• VISTA Enhancers (1745 human enhancers downloaded on December 21, 2015) human elements: (https://enhancer.lbl.gov/)
• Super enhancers: (https://asntech.org/dbsuper/)
• Blacklisted CpGs: (http://genome.ucsc.edu/cgi-bin/hgFileUi?db=hg19&g=wgEncodeMapability)
• chromHMM 18-state maps and 15-state maps: downloaded from Roadmap Epigenomics Data Portal, https://egg2.wustl.edu/roadmap/web_portal/
• NH-A EZH2 CHIP-seq: [GEO: GSM1003532]
• 450k and 850k array probe locations: Locations of Illumina HumanMethylation450 BeadChip probes (Infinium HumanMethylation450K v1.2) were downloaded from https://support.illumina.com/array/array_kits/infinium_humanmethylation450_beadchip_kit/downloads.html ("HumanMethylation450 v1.2 Manifest File (CSV Format)"). Locations of Illumina MethylationEPIC BeadChip probes (850K array) were downloaded from https://support.illumina.com/array/array_kits/infinium-methylationepic-beadchip-kit/downloads.html ("Infinium Methylation EPIC v1.0 B4 Manifest File (CSV Format)")
• Tumor suppressor genes: (https://bioinfo.uth.edu/TSGene/)
• TCGA EAC, normal endometrium, and GBM RNA-seq data (level-3, RPKM) and clinical metadata of EAC, GBM, and their matched-control samples were downloaded from the Genomic Data Commons Data Portal (https://portal.gdc.cancer.gov/).
• Adipose (subcutaneous, visceral) and brain (frontal cortex) RNA-seq: GTEx.
• Apoptosis gene list: KEGG72 (Entry: hsa04210, n=140)
• ChIA-PET HeLa RNAPol2: ENCODE data portal (https://www.encodeproject.org/).
• Processed adipose ChIP-seq data (bigWig, H3K4me1, H3K4me3, and H3K27ac) were downloaded from the ENCODE data portal (https://www.encodeproject.org/).
• A549 TCF12 ChIP: ENCODE data portal (https://www.encodeproject.org/).
• ChIP-PET K562 RNAPol2: ENCODE data portal (https://www.encodeproject.org/).
• H3K27ac and H3K4me1 Peaks in Adult and Fetal Brain Samples: We obtained H3K4me1 peak files from Roadmap (downloaded from Roadmap Epigenomics Data Portal (https://egg2.wustl.edu/roadmap/web_portal/)) for the following samples: E053, E054, E070, E081, E082, E125 (fetal brain); and E067, E068, E069, E071, E072, E073, E074 (adult brain); as well as H3K27ac narrow peak files from Roadmap (downloaded from Roadmap Epigenomics Data Portal (https://egg2.wustl.edu/roadmap/web_portal/)) for the following samples: E125 (fetal brain); and E067, E068, E069, E071, E072, E073, E074 (adult brain).
• RepeatMasker annotations were downloaded from the UCSC Genome Browser: (https://hgdownload.soe.ucec.edu/download.html)
• WGBS data (thymus, ovary, pancreas, lung, mid-frontal cortex, brain germinal matrix, frontal cortex neuron, frontal cortex glia, atrium, sigmoid colon, colon tumor, colorectal cell line HCT116, breast myoepithelial, breast cancer HCC1954, liver, HepG2) was downloaded from the Roadmap data portal (http://www.roadmapepigenomics.org/) and the ENCODE data portal (https://www.encodeproject.org/).
• U87 H3K4me3 ChIP-seq [GEO: GSM2634761]
• TCGA Infinium 450k array probe data for all available cancer types: (https://portal.gdc.cancer.gov/)
• Normal glia RNA-seq: [GEO: GSE41826]
• TCGA methylation array data: https://gdc.cancer.gov/

Field-specific reporting

Please select the one below that is the best fit for your research. If you are not sure, read the appropriate sections before making your selection.

☒ Life sciences ☐ Behavioural & social sciences ☐ Ecological, evolutionary & environmental sciences

For a reference copy of the document with all sections, see nature.com/documents/nr-reporting-summary-flat.pdf

Life sciences study design

All studies must disclose on these points even when the disclosure is negative.

Sample size	Differentially methylated regions were analyzed from previous studies which included complete DNA methylomes generated for a total of 5 GBM samples, 2 normal frontal cortex brain samples, 3 EAC samples, and 1 normal endometrial sample pooled from 10 healthy individuals. Validation of methylation alterations using TCGA array-based datasets provides confidence in the methylation changes observed in this set of samples (see Supplementary Figures 2 and 3).
Data exclusions	No data were excluded.
Replication	As described above, there were 5 GBM samples, 3 EAC samples, and 2 normal brain samples analyzed. The only sample without a replicate was the normal endometrial sample. However, validation of methylation alterations using TCGA array-based datasets provided confidence in the methylation changes observed (see Supplementary Figure 2 and 3).
Randomization	NA - publicly available data were used.
Blinding	NA - publicly available data were used.

Reporting for specific materials, systems and methods

We require information from authors about some types of materials, experimental systems and methods used in many studies. Here, indicate whether each material, system or method listed is relevant to your study. If you are not sure if a list item applies to your research, read the appropriate section before selecting a response.

Materials & experimental systems

n/a	Involved in the study
☒	☐ Antibodies
☒	☐ Eukaryotic cell lines
☒	☐ Palaeontology and archaeology
☒	☐ Animals and other organisms
☒	☐ Human research participants
☒	☐ Clinical data
☒	☐ Dual use research of concern

Methods

n/a	Involved in the study
☒	☐ ChIP-seq
☒	☐ Flow cytometry
☒	☐ MRI-based neuroimaging
